# MethylModes: computationally efficient detection of multimodal distributions in DNA methylation data

**DOI:** 10.1093/bioinformatics/btag045

**Published:** 2026-01-22

**Authors:** T Sophia Luo, Jonathon LeFaive, John Dou, Kelly M Bakulski, Erin B Ware, Matthew Zawistowski

**Affiliations:** Department of Biostatistics, University of Michigan School of Public Health, Ann Arbor, MI 48109, United States; Department of Biostatistics, University of Michigan School of Public Health, Ann Arbor, MI 48109, United States; Department of Epidemiology, University of Michigan School of Public Health, Ann Arbor, MI 48109, United States; Department of Epidemiology, University of Michigan School of Public Health, Ann Arbor, MI 48109, United States; Institute for Social Research, Survey Research Center at the University of Michigan, Ann Arbor, MI 48106, United States; Department of Biostatistics, University of Michigan School of Public Health, Ann Arbor, MI 48109, United States

## Abstract

**Summary:**

MethylModes is an R package and Shiny application to identify multimodal distributions in human DNA methylation at individual CpG sites. Multimodal distributions, which can be the result of nearby genetic variation, environmental exposures, or assay artifacts, are susceptible to confounding and important to identify for methylation analysis. MethylModes is easily incorporated into existing quality control pipelines of array-based DNA methylation data. The underlying algorithm uses kernel smoothing of probe-level data to locate the number and location of peaks. The algorithm can be parallelized across probes for efficient implementation at genome-scale. We provide a case study implementation of MethylModes in the Health and Retirement Study as well as the Airwave Health Monitoring Study.

**Availability and implementation:**

MethylModes is available on GitHub at https://github.com/lutiffan/methylModes as an R package wrapping an R Shiny application. We include a toy dataset to validate installation. The codebase is also published on Zenodo at https://doi.org/10.5281/zenodo.17448517.

## 1 Introduction

DNA methylation is a dynamic biological process in which a methyl group is chemically bonded to DNA (Moore *et al.* 2013), usually at the cytosine nucleotide of a cytosine-phosphate-guanine (CpG) motif (Schultz *et al.* 2015). DNA methylation plays an essential role in physiological development by regulating gene expression (Moore *et al.* 2013). Changes in DNA methylation patterns are associated with adverse health outcomes (Robertson 2005), motivating epigenetic studies to investigate links between DNA methylation, genetic variation, environmental exposures, and disease. Array-based methylation assays, such as Illumina’s Infinium MethylationEPIC BeadChip, are efficient for quantifying DNA methylation levels across the methylome in large cohorts (Illumina Support, https://support.illumina.com/). However, the assays are susceptible to confounding by biological factors such as genetic variation at or near the probe binding site as well as technical artifacts such as cross-hybridization of the probe to multiple locations in the genome (Naeem *et al.* 2014, McCartney *et al.* 2016). These issues can result in probes with multimodal methylation values, which can violate regression assumptions, produce false associations, and reduce power to detect true associations. Empirical methods to identify multimodal CpG probes are therefore an important quality control procedure for a DNA methylation analysis.

Individual-level methylation at a specific CpG site is commonly quantified using the beta value, the ratio of methylated to total (unmethylated plus methylated) intensities in the biological sample. Beta values range between 0, indicating complete lack of methylation at all copies of the CpG site in the individual’s biological sample, to 1, indicating all CpG sites are methylated (Du *et al.* 2010). The distribution of beta values at a given CpG site across all samples provides insight into variation in methylation driven by biological factors as well as potential artifacts. Most CpG probes have unimodal distributions. A well-known cause of multimodality is a single nucleotide polymorphism (SNP) at the CpG site, which creates a trimodal distribution with distinct peaks corresponding to genotypes of the SNP. Methylation quantitative trait loci (mQTLs) and technical artifacts such as cross-hybridization can result in subtler, irregular forms of multimodality. We developed MethylModes to robustly identify multimodality in beta value distributions using an intuitive algorithm that avoids stringent modeling assumptions.

MethylModes builds on existing approaches. Andrews *et al.* proposed *gaphunter* to identify multimodal beta distributions based on “gaps” between discrete clusters of observed beta values (Andrews *et al.* 2016), a strong requirement that results in reduced ability to identify distinct clusters as sample sizes increase (Hu and Li 2018). *MethylToSNP* uses a clustering algorithm to identify probes with a SNP at or adjacent to the CpG site (LaBarre *et al.* 2019). Other methods allow for the identification of subtler multimodal distributions. Hu and Li proposed a Gaussian mixture model clustering (GMMC) approach. Xu *et al.* implemented a peak detection algorithm based on kernel density estimation (KDE) as part of the *ENMix* R package, which uses a sliding-window approach to identify local maxima (Xu *et al.* 2016). Figure 3, available as supplementary data at *Bioinformatics* online, illustrates the classification performed by *gaphunter* and GMMC. MethylModes uses a KDE approach to smooth the empirical beta value histograms and identify stationary points on the curve, then applies denoising steps to differentiate local maxima from distinct modes. MethylModes can be run on all CpG sites in an efficient parallelized manner or restricted to specific genomic regions. We implemented a user-friendly Shiny app to allow visual inspection of the multimodal calls. The app includes annotations for CpG sites on the Illumina Infinium HumanMethylation450 BeadChip, EPIC v1.0, and EPIC v2.0 arrays to cross-tabulate modality with various probe features, including relationships to CpG islands, SNPs under probes, and hypo- and hypermethylation status. MethylModes results can be exported as .csv files.

## 2 Materials and methods

### 2.1 Parameters and filtering

MethylModes has two peak detection parameters: *peakDistance* and *proportionSample*. *peakDistance* specifies the minimum distance required between two adjacent peaks. *proportionSample* specifies the minimum proportion of the sample size captured within the boundaries of the peak. The MethylModes app has optional thresholds for classifying hypo- and hyper-methylated sites, which are characterized by the probe-level mean and variance of beta values across the sample.

We applied a post-hoc filtering step based on the proportion of samples contained in the secondary peak (in MethylModes results, column “proportionSample_2”). Intuitively, sites for which the secondary peak contains a large proportion of samples represent unambiguous multimodality rather than noise. The proportion of samples in the secondary peak also provides a convenient means by which to rank CpG sites based on confidence in multimodality.

### 2.2 Peak detection algorithm

MethylModes uses the KDE method implemented in base R to infer the locations of distinct modes in distributions of beta values at individual probes (R Core Team 2024, https://www.r-project.org/). Figure 3, available as supplementary data at *Bioinformatics* online, provides illustration of algorithm steps and comparison to previous methods.

1. Smoothing of the empirical beta distribution: perform KDE using the stats::density() function.
2. Detection of local maxima and minima:
  - Approximate the slope by calling base::diff() on fitted KDE values.
  - Calculate a logical vector: if slope > 0, then TRUE, else FALSE.
  - Determine locations of stationary points by identifying indices where the logical vector switches from sequences of TRUE to FALSE and vice versa.
  - Candidate peaks are defined as locations where the slope changes from positive to zero or negative, i.e. a local maximum.
3. Classification of modality:
  - If only one candidate peak was identified, classify the CpG site as unimodal.
  - Otherwise, proceed to noise filtering steps.
    - Calculate the proportion of the sample contained within each candidate peak. If a peak contains less than *proportionSample* of the sample and one of its bounding minima is equal to zero, it is considered noise and discarded.
    - Adjacent peaks that are within *peakDistance* of each other are considered to be a group of local maxima that represent the same mode and are merged.
    - Re-calculate sample proportion in merged peaks. If the combined proportion is below *proportionSample*, then discard it.
    - If one peak remains, classify the CpG site as unimodal. Otherwise, classify the site as multimodal.

Figure 2, available as supplementary data at *Bioinformatics* online, contains pseudocode for the above steps.

### 2.3 Software input

**Figure 1:**
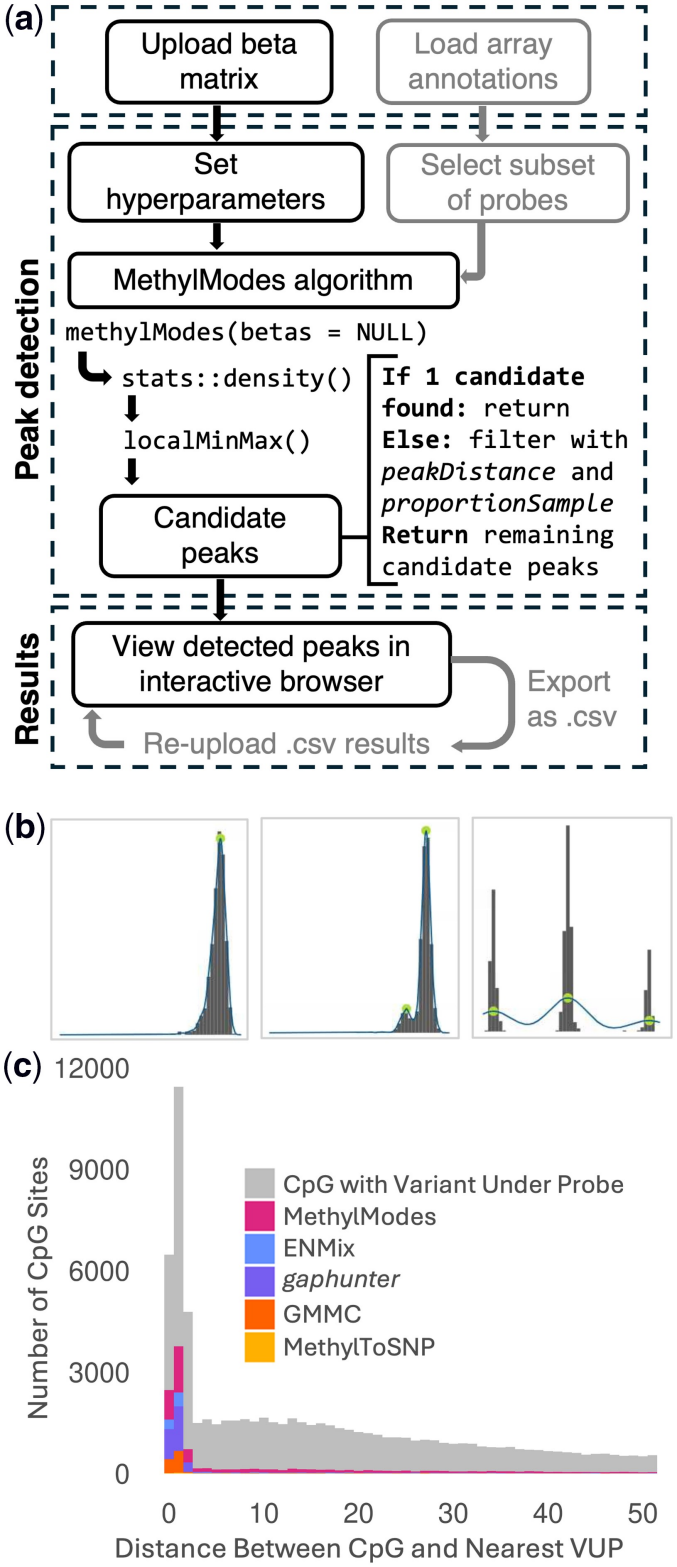
(a) Overview of full MethylModes workflow, with optional steps in grey; (b) Example visualizations of MethylModes results with identification of uni- or multimodality. Left to right: unimodal, multimodal (two peaks), and multimodal (three peaks); (c) Comparison of CpG sites with a variant under the probe (MAF > 0.01) identified by each method as multimodal in the HRS dataset as a function of distance between the CpG site and nearest SNP. MethylModes parameters were set to: *proportionSample *= 0.01, *peakDistance *= 0.10. MethylModes results were filtered to remove probes with a secondary peak smaller than 4% of the sample. All other methods were run using author-recommended parameters.

The input for MethylModes is an R matrix of methylation beta values. Accepted formats include .csv and .RDS files. We recommend running MethylModes on methylation data that has already undergone standard quality control steps including removal of .idat file irregularities, sex mismatches, and technical replicates. MethylModes returns a .csv file with columns containing probe name, mean beta value across samples, MethylModes-inferred modality, peak location along with corresponding sample proportions and variances within each peak, and flags indicating hypo- or hyper-methylation status. Figure 1a summarizes the Shiny app workflow. A toy dataset is provided with the R package to demonstrate file format and to validate package installation. The MethylModes workflow is provided in Fig. 1, available as supplementary data at *Bioinformatics* online.

### 2.4 Real-world use cases: the health and retirement study and the airwave health monitoring study

The Health and Retirement Study (HRS) is a nationally representative, longitudinal study of Americans over age 50 (Juster and Suzman 1995, Sonnega *et al.* 2014). Blood DNA methylation was measured using the Illumina Infinium MethylationEPIC v1.0 BeadChip for 4018 HRS participants who participated in the 2016 Venous Blood Study. The dataset and methylation quality control steps have been previously described (https://hrs.isr.umich.edu/sites/default/files/biblio/HRS2016VBSDD_1.pdf, https://hrs.isr.umich.edu/sites/default/files/genetic/HRS_DNAm_OCT2023.pdf) (Crimmins *et al.* 2016, Faul *et al.* 2023). We performed further quality control by removing 95 431 probes with detection p-value < 0.0001 using the *ewastools* R package (Murat *et al.* 2020). An additional 41 071 probes were removed for cross-reactivity (Zhou *et al.* 2017). Twenty-four samples were excluded due to low signal intensity, control probe failures, or high genetic relatedness. A total of 3994 samples and 729 529 probes were analyzed in this study. The Airwave Health Monitoring Study consists of British police officers and staff (Robinson *et al.* 2020) obtained from the *recountmethylation* R package (Maden *et al.* 2023). Blood DNA methylation was measured using the Infinium HumanMethylation EPIC BeadChip for 1110 participants. We analyzed the available data based on previously described quality control steps (https://police-health.org.uk/sites/default/files/2024-06/Annex-K-DNA-Methylation.pdf) (Karimi and Chadeau-Hyam 2018).

The EPIC v1.0 manifest includes 729 529 probes whose hybridization sequence overlaps with at least one documented SNP, of which 74 417 include at least one SNP with minor allele frequency (MAF) > 0.01. We refer to these SNPs within probe hybridization sequences as variants under the probe (VUP). Because CpG sites with VUPs often have a multimodal beta value distribution, we defined the sensitivity of a method as the percentage of probes overlapping variants with MAF > 0.01 that were detected by the method (Price *et al.* 2013, Naeem *et al.* 2014). Default MethylModes parameter values were chosen using a grid search that balanced detection of probes with VUPs with sensitivity to noise (Fig. 4, available as supplementary data at *Bioinformatics* online). We chose parameter values of *proportionSample *= 0.01 and *peakDistance *= 0.10. Two additional parameter combinations were tested (Fig. 8, available as supplementary data at *Bioinformatics* online). A post-processing filter discarding multimodal calls whose secondary peak was smaller than 4% of the sample was applied, where the cutoff was determined by visual inspection (Fig. 6, available as supplementary data at *Bioinformatics* online). GMMC was implemented using the *mclust* R package and the post-processing parameters specified by Hu *et al.* with the user-specified parameter of OUT_CUTT set to 0.9 to filter out typical unimodal distributions (https://cran.r-project.org/web/packages/mclust/index.html) (Hu and Li 2018, Fraley *et al.* 2024). *gaphunter* was run using its author-recommended parameters. We ran the *nmode* function in *ENMix* using its default parameters. *MethylToSNP* software was found to be limited to datasets containing fewer than 2000 samples; to run it on HRS data, we created a copy with a minor modification removing the limitation.

## 3 Results and discussion

In the Health and Retirement Study dataset, MethylModes classified 10 967 of 73 645 (14.9%) autosomal CpG sites with VUPs with MAF > 0.01 as multimodal (Fig. 7, available as supplementary data at *Bioinformatics* online). Figure 1b provides examples of modality classifications. Of these, 5806 (52.9%) were not identified by other methods. *ENMix* identified 5132 (7.0%) of the VUP sites, *gaphunter* identified 3716 (5.0%), GMMC identified 1366 (1.9%), and *MethylToSNP* identified 81 (0.1%). Overlap with MethylModes was greatest with *ENMix* (90.9%) followed by GMMC (85.7%) and then *gaphunter* (60.1%) (Figs 7 and 8, available as supplementary data at *Bioinformatics* online). MethylModes demonstrated greater sensitivity to identify subtle multimodal patterns in which the VUP was further from the CpG site (Fig. 1c). Of probes called by *gaphunter* but not MethylModes, 1284 (86.6%) are probes initially called as multimodal by MethylModes but were discarded based on the filter requiring secondary peaks to contain at least 4% of samples. The low overlap with *MethylToSNP* is unsurprising, given the different objectives of the methods: *MethylToSNP* aims to find probes with SNPs 0–1 bp from the CpG site, using an algorithm that targets a “three-tier” pattern defined by two large gaps between three distinct clusters (LaBarre *et al.* 2019). Of the 79 probes found by *MethylToSNP* and not MethylModes, 12 had been filtered out of MethylModes results using the secondary peak; the remaining probes were visualized in the MethylModes Shiny app and found to have unimodal distributions. While the extent of overlap with calls from other methods changes with different MethylModes parameter values, it consistently remains the most sensitive method (Fig. 8, available as supplementary data at *Bioinformatics* online).

We ran all methods on the Airwave dataset (Fig. 9, available as supplementary data at *Bioinformatics* online). Results were consistent with that of a downsampled version of the HRS dataset (Fig. 10, available as supplementary data at *Bioinformatics* online). Namely, MethylModes identified the largest number of CpGs with VUP as multimodal, followed by *gaphunter*, highlighting the known sensitivity of *gaphunter* to smaller sample sizes.

## 4 Conclusion

MethylModes is an intuitive peak detection algorithm that contributes to the power of downstream epigenetic analyses. It provides a balance between computational efficiency and improved multimodal detection over existing methods (Fig. 11, available as supplementary data at *Bioinformatics* online) and is readily incorporated into existing pipelines. The MethylModes Shiny app is a novel tool for visual inspection of multimodal beta distributions. Given the subjective nature of multimodality, the app is a crucial accompaniment to interpret results and fills a critical gap in quality control of high-throughput methylation data. We assessed performance of MethylModes and other methods in the large and ancestrally diverse HRS dataset and confirmed performance in an independent cohort.

A key challenge is objectively defining multimodal distributions. Multimodality exists on a spectrum, ranging from distributions containing distinct and prominent peaks with clear separation to subtle fluctuations that can be attributable to noise. The primary goal of MethylModes is to distinguish unimodality versus multimodality rather than discerning the exact number of peaks since this aligns with analysis considerations. As with previous methods, inferred modality in MethylModes is sensitive to parameter choices (Figs 5 and 8, available as supplementary data at *Bioinformatics* online). Although MethylModes reduces subjectivity in peak detection by imposing numeric thresholds, we cannot avoid the inherent limitation in the lack of a formal definition for multimodality. Since the degree of multimodality is driven by a variety of biological and technical factors, the ultimate decision of whether to exclude a given probe from analysis is left to the investigator based on study goals. MethylModes empowers researchers to make informed quality control decisions for methylation array data.

## Supplementary Material

btag045_Supplementary_Data

## Data Availability

The data from the Health and Retirement Study used in this article was accessed from The National Institute on Aging Genetics of Alzheimer's Disease Data Storage Site (NIAGADS-DSS), accession number NG00153 (https://dss.niagads.org/datasets/ng00153/). The Airwave Health Monitoring Study data was accessed via the *recountmethylation* R package, and is also accessible via the Gene Expression Omnibus database, accession number GSE147740 (https://www.ncbi.nlm.nih.gov/geo/query/acc.cgi?acc=GSE147740).
