# Supplementary material for: MethylModes: computationally efficient detection of multimodal distributions in DNA methylation data
Source: Bioinformatics. 2026 Jan 22;42(2):btag045. doi: 10.1093/bioinformatics/btag045 (PMC12883086; doi:10.1093/bioinformatics/btag045)
Supplement: btag045_Supplementary_Data [file btag045_supplementary_data.docx]

**Supplementary Material**


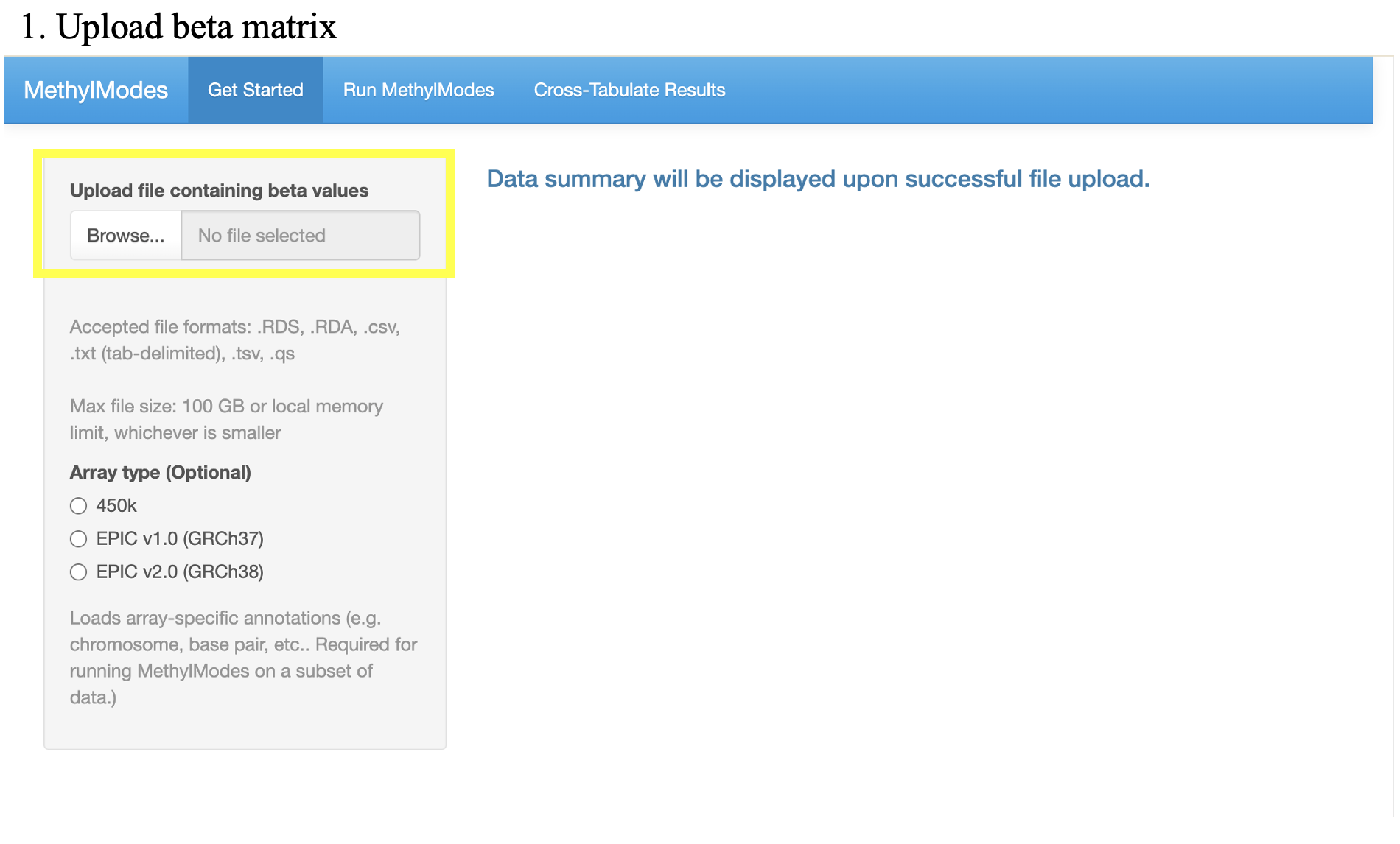


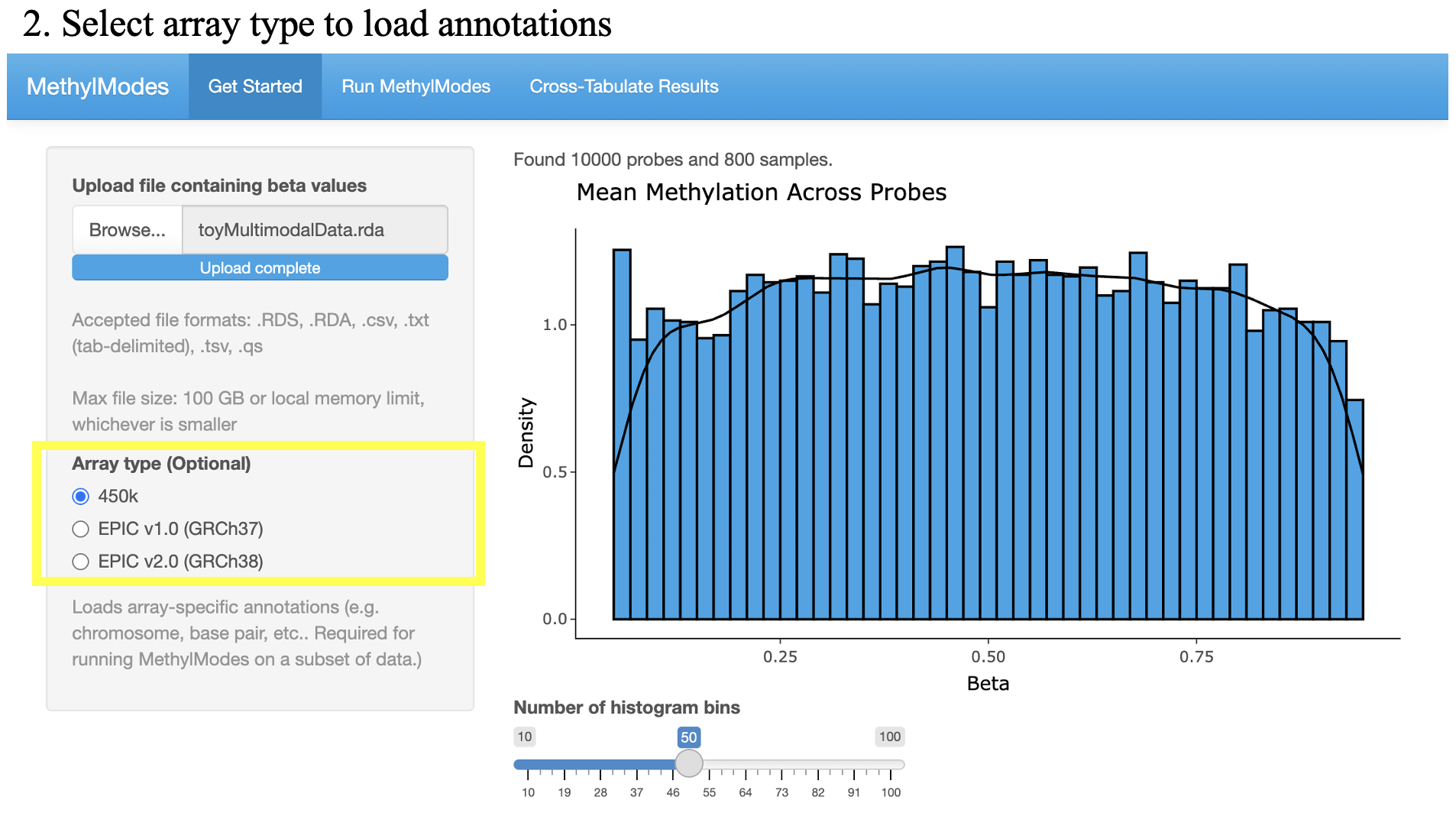


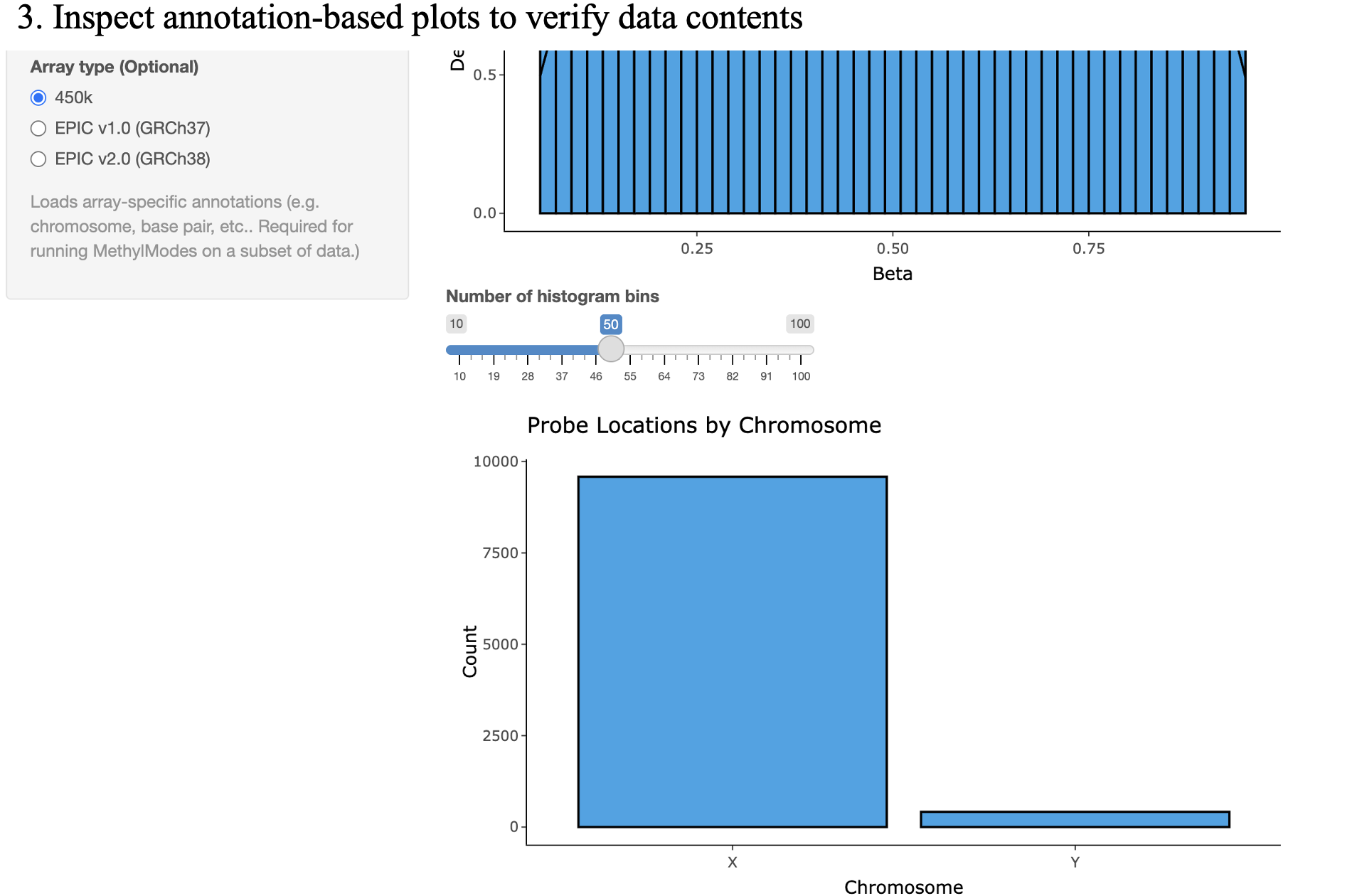


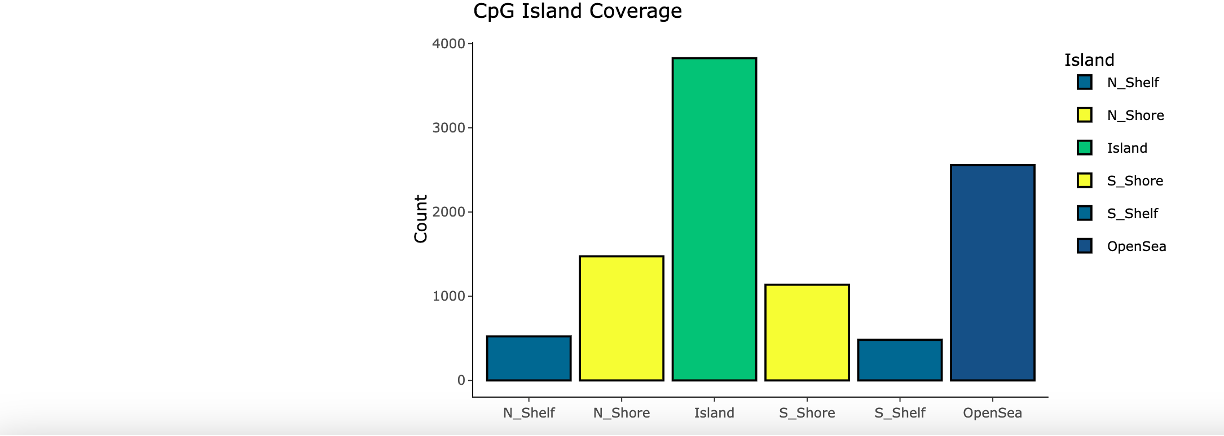


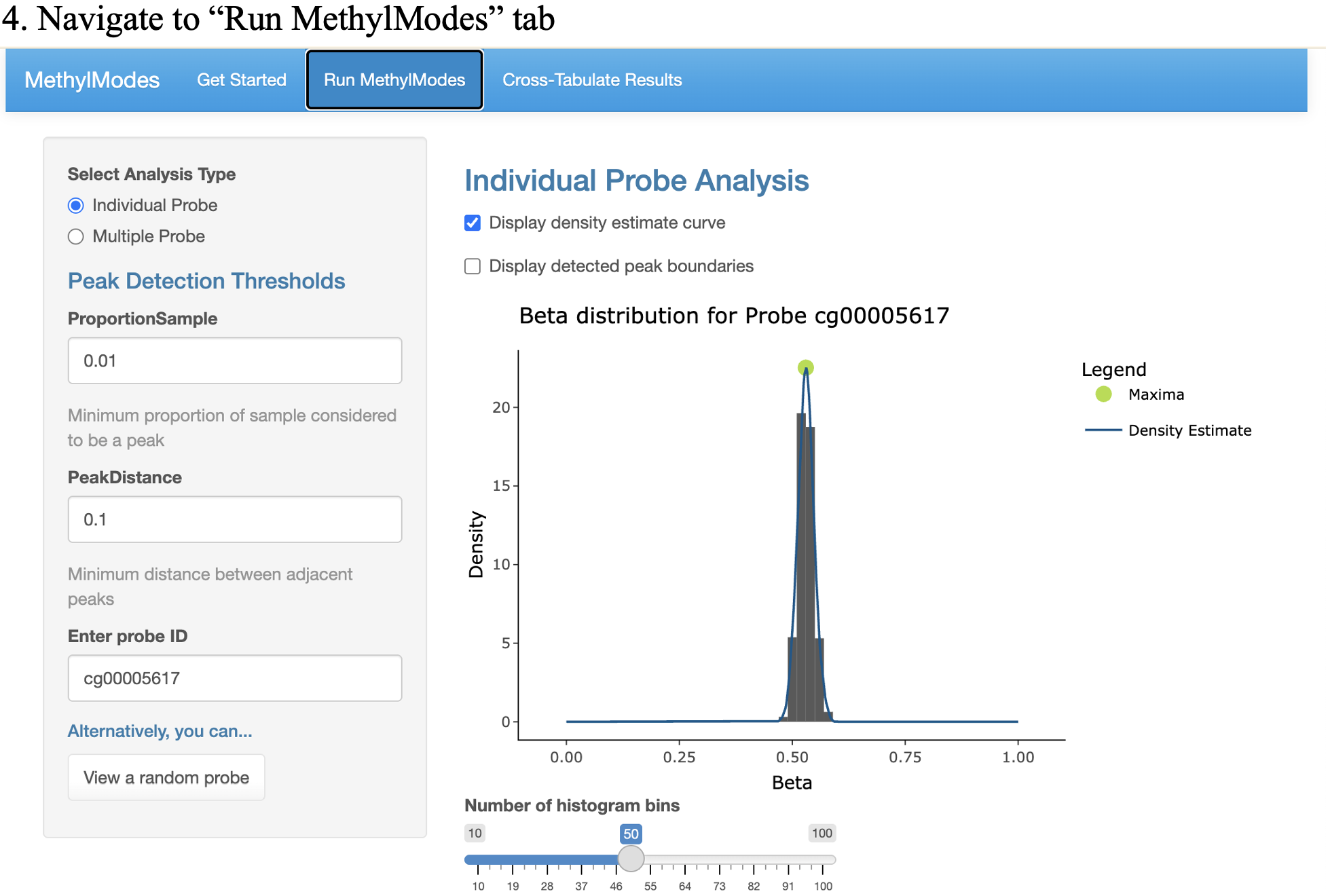


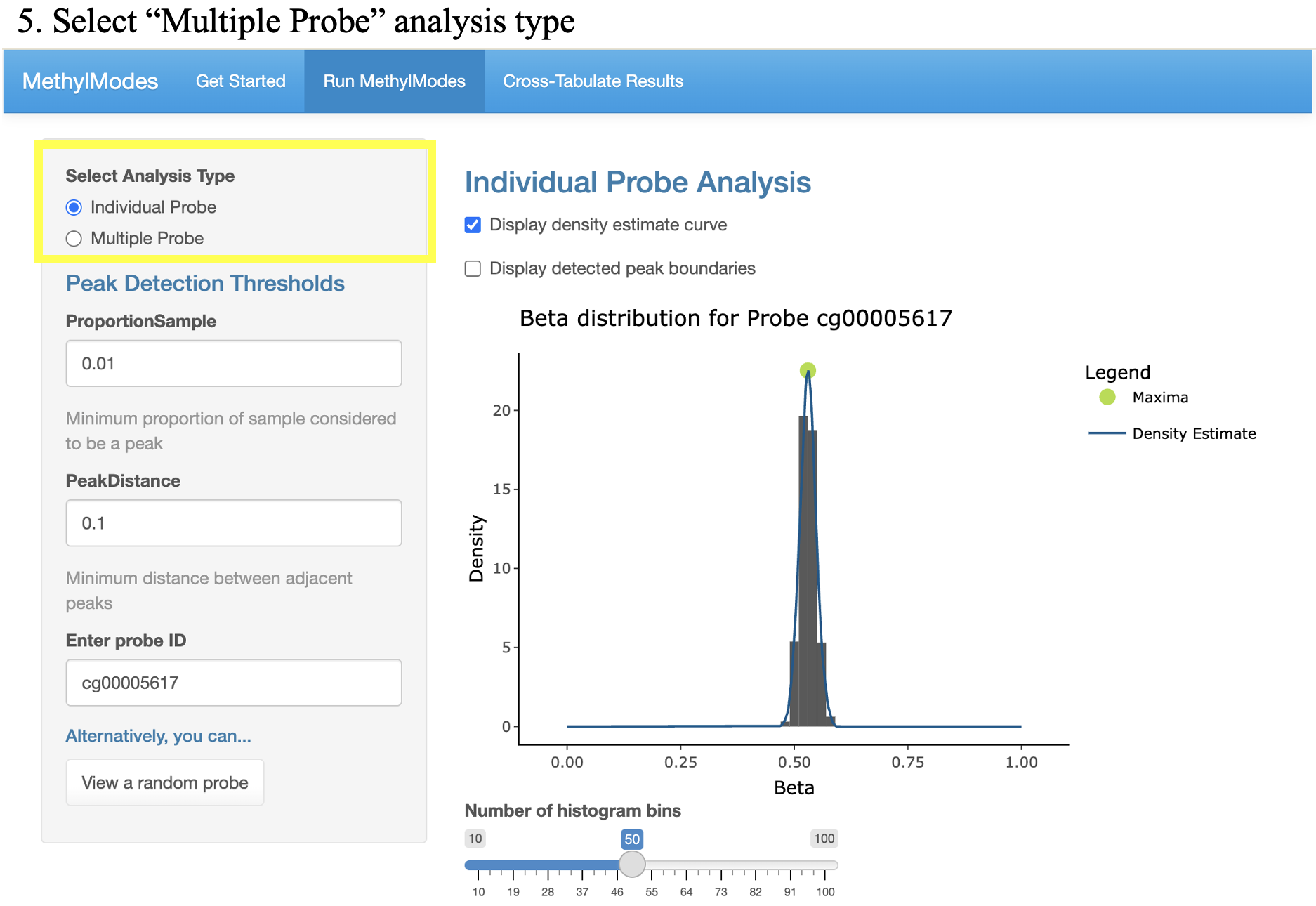


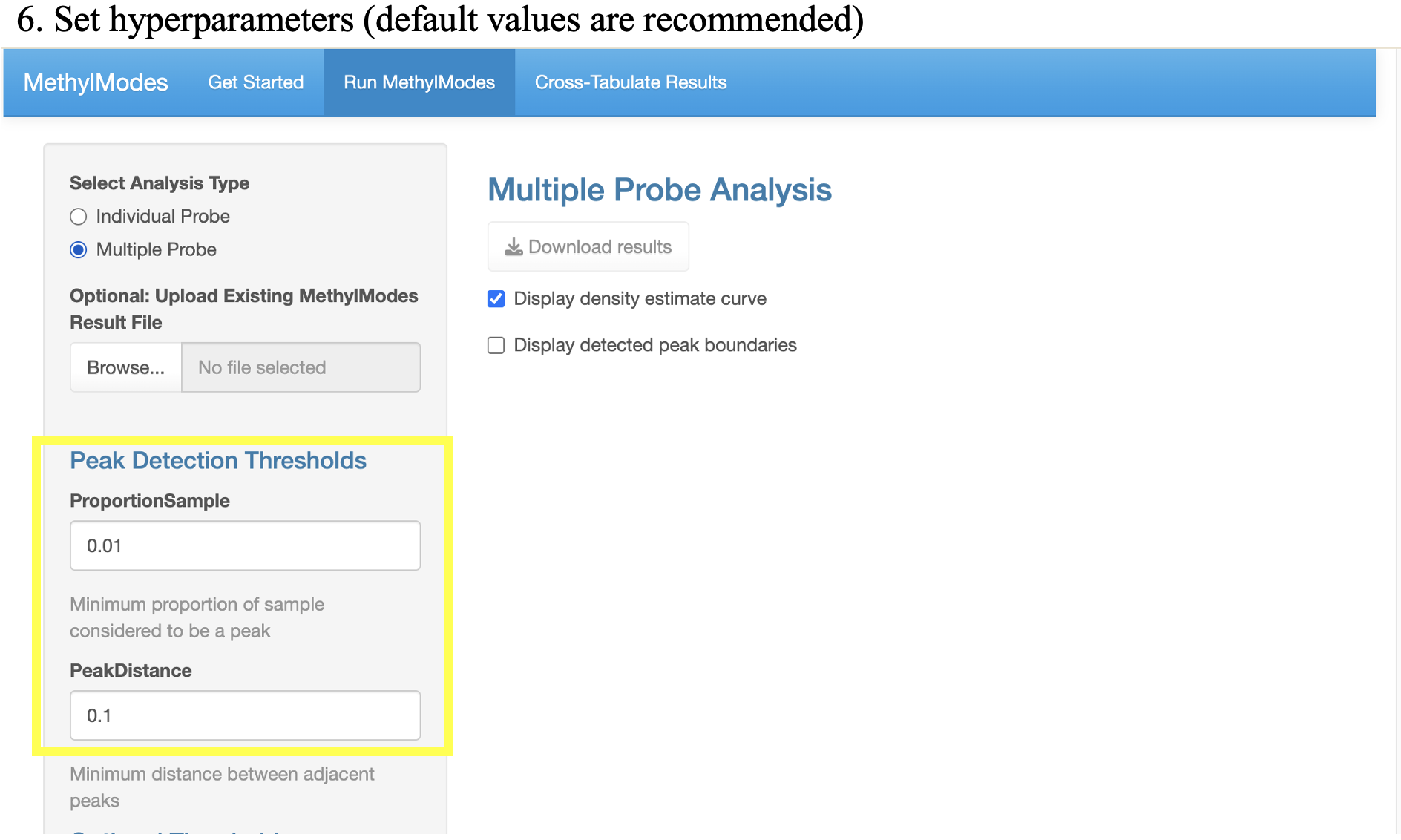


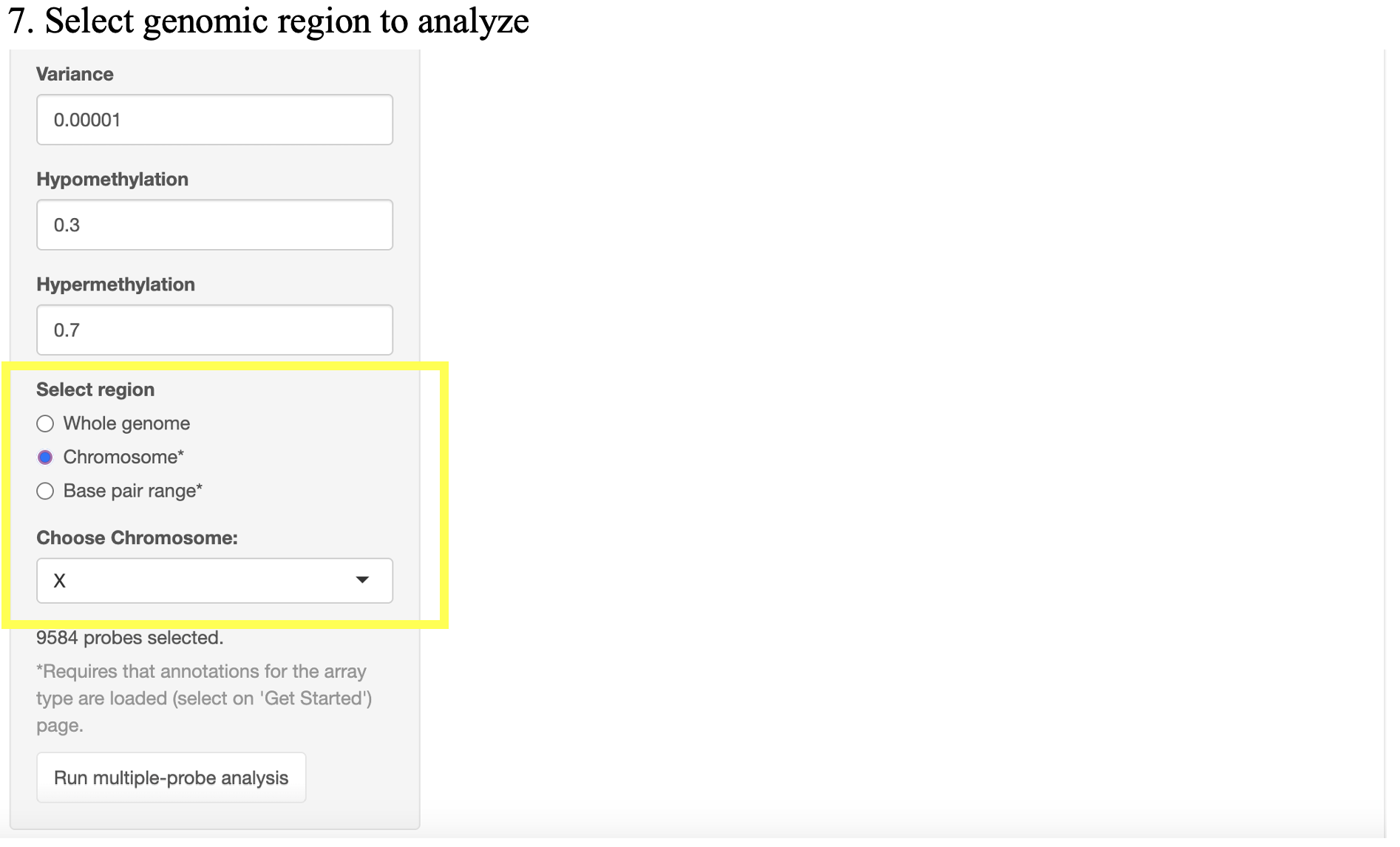


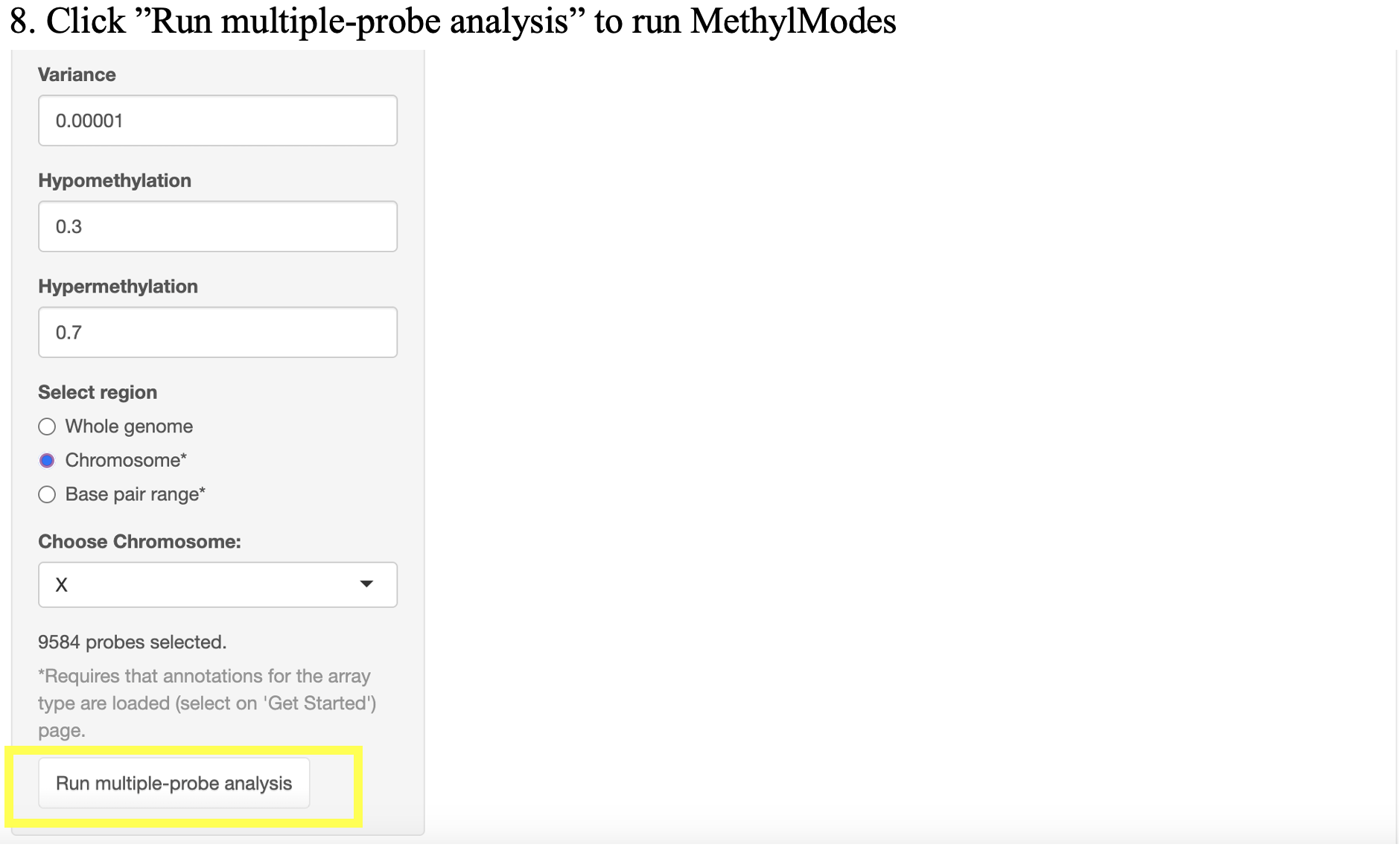


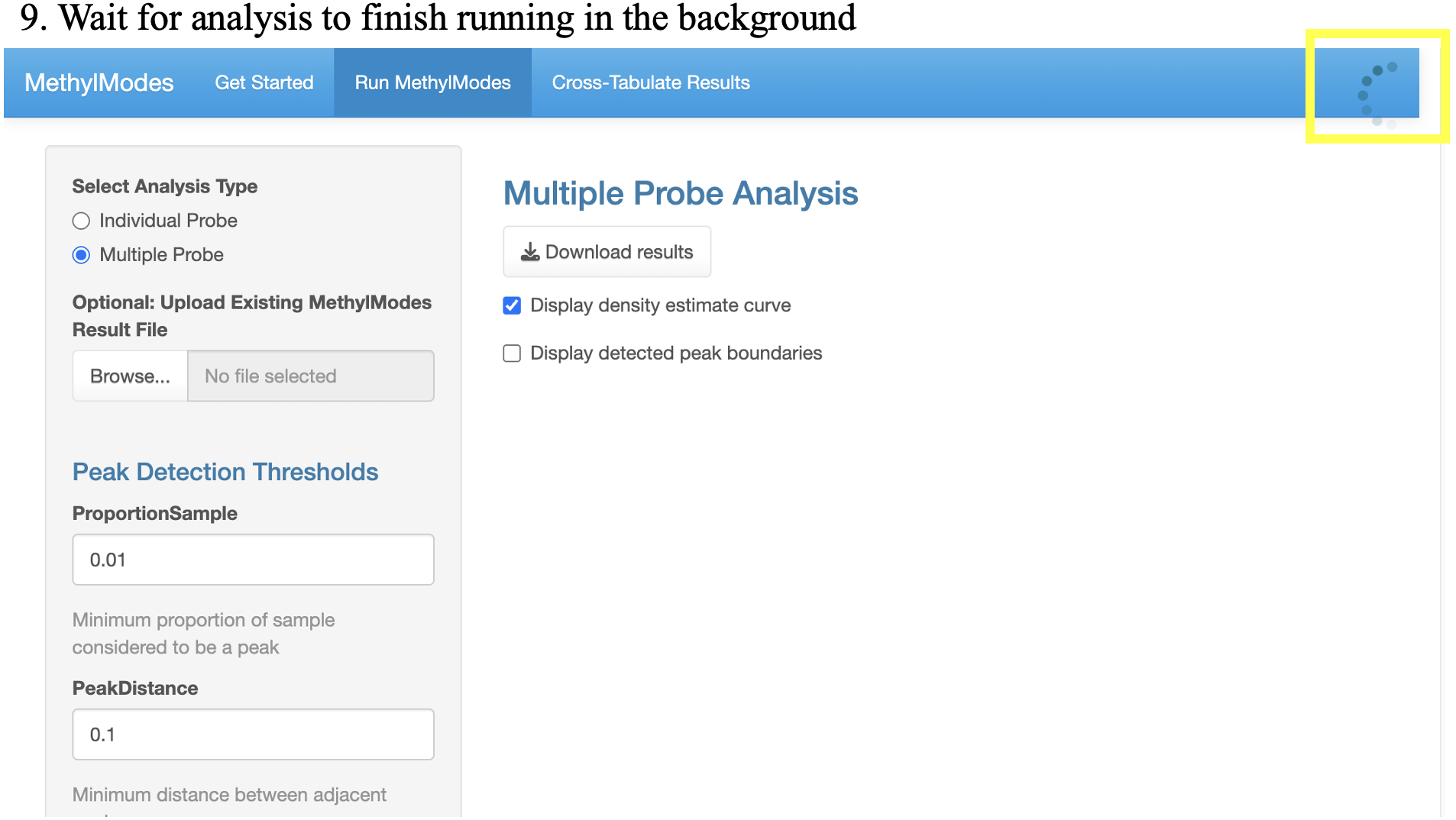


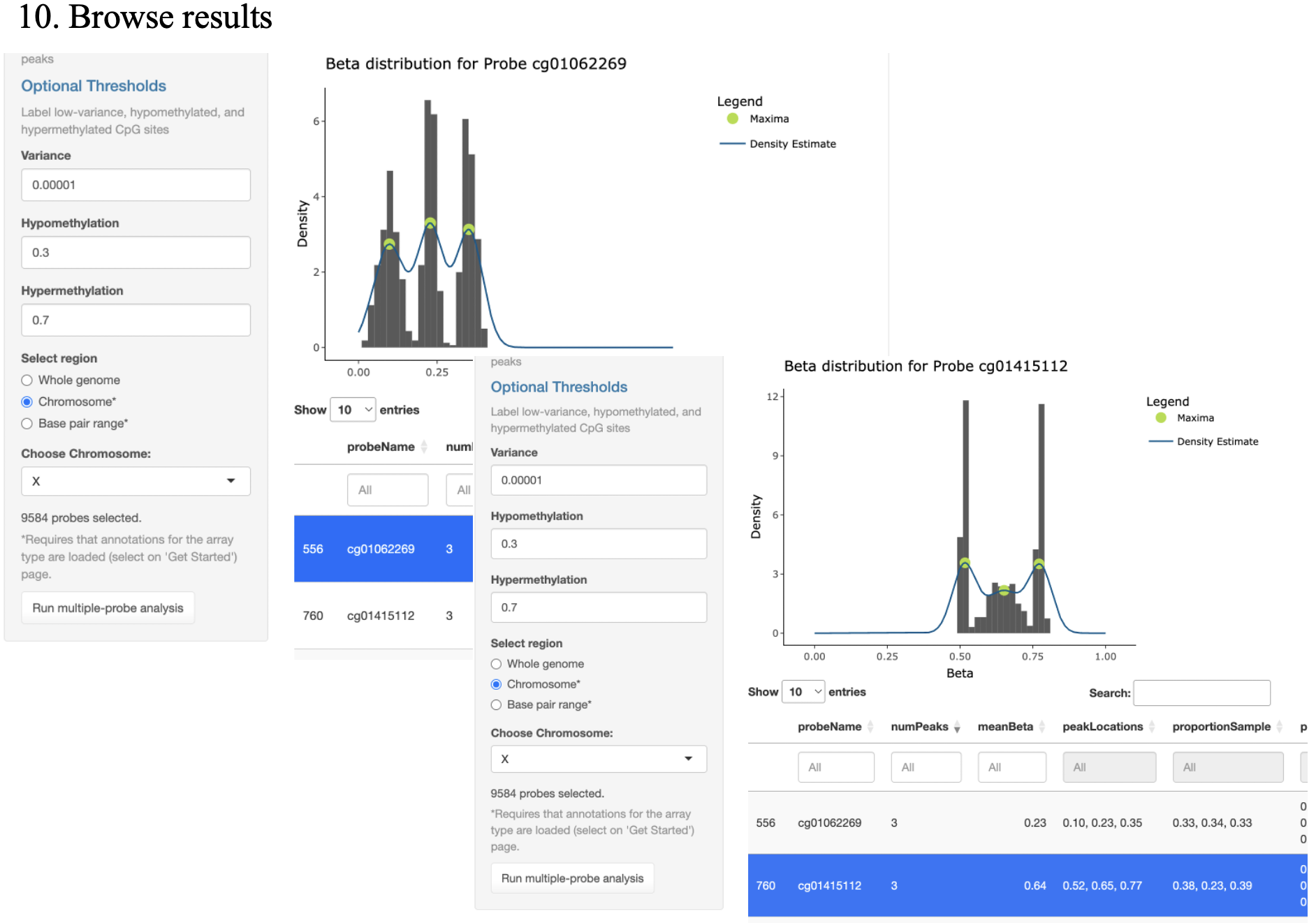


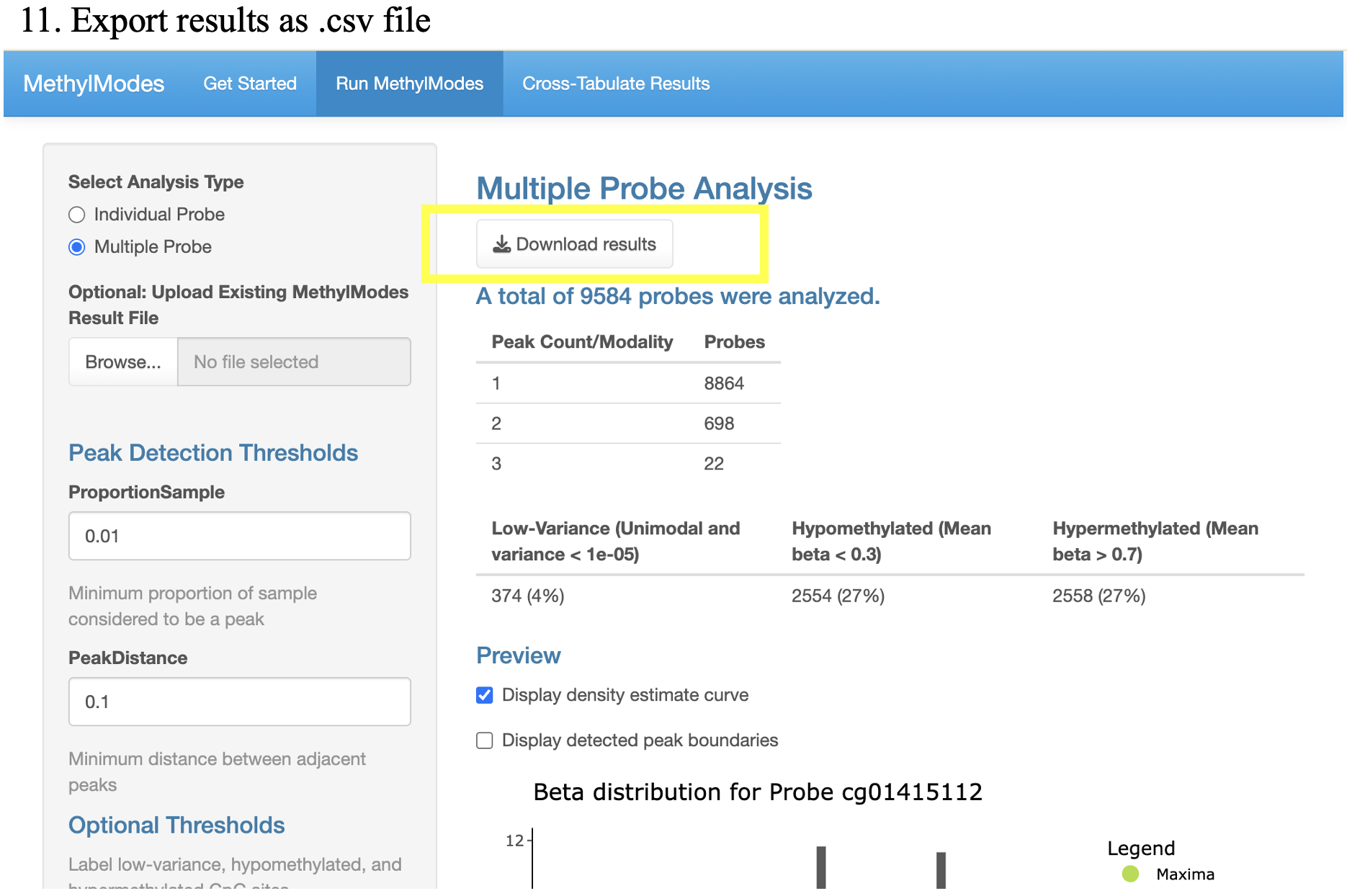


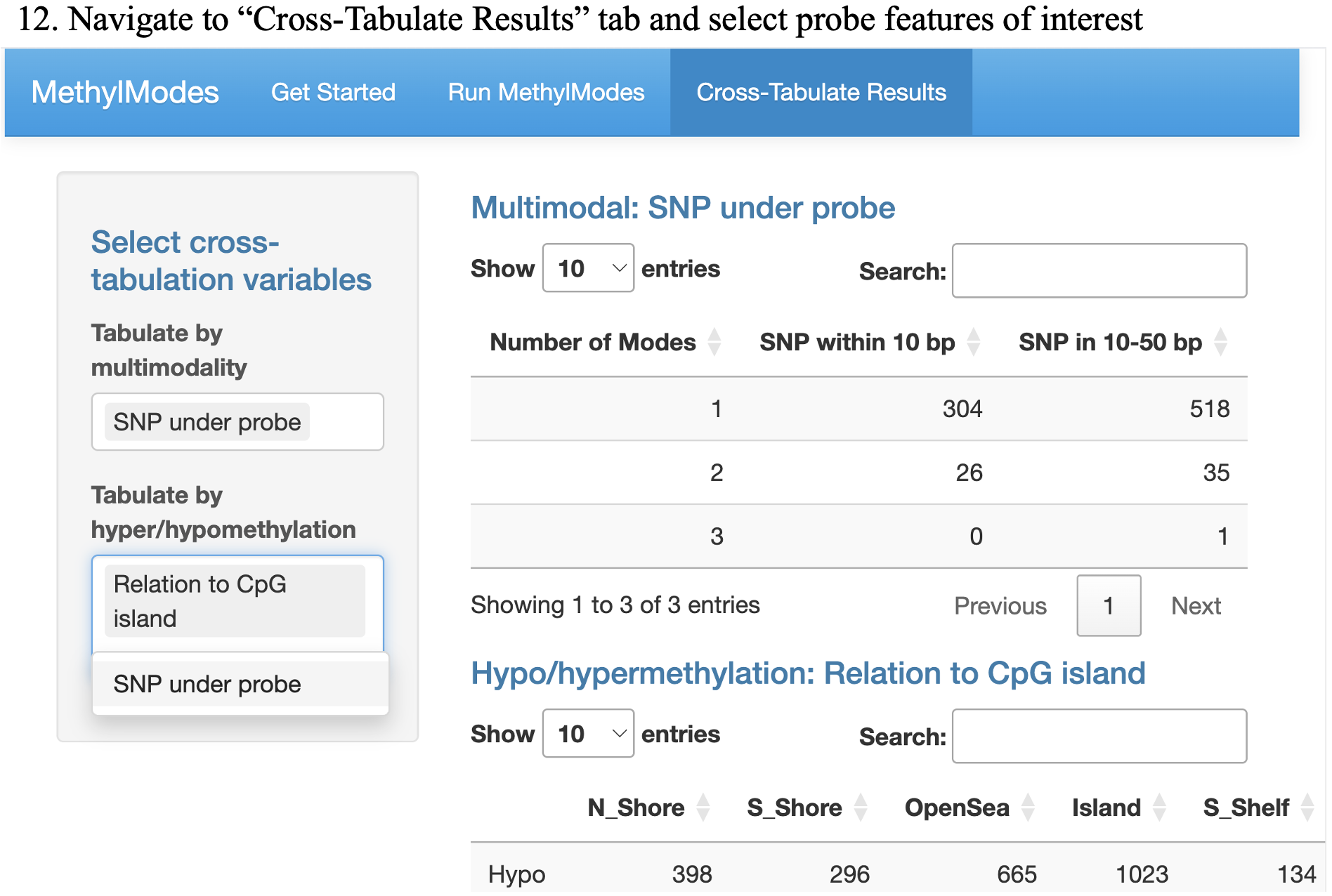


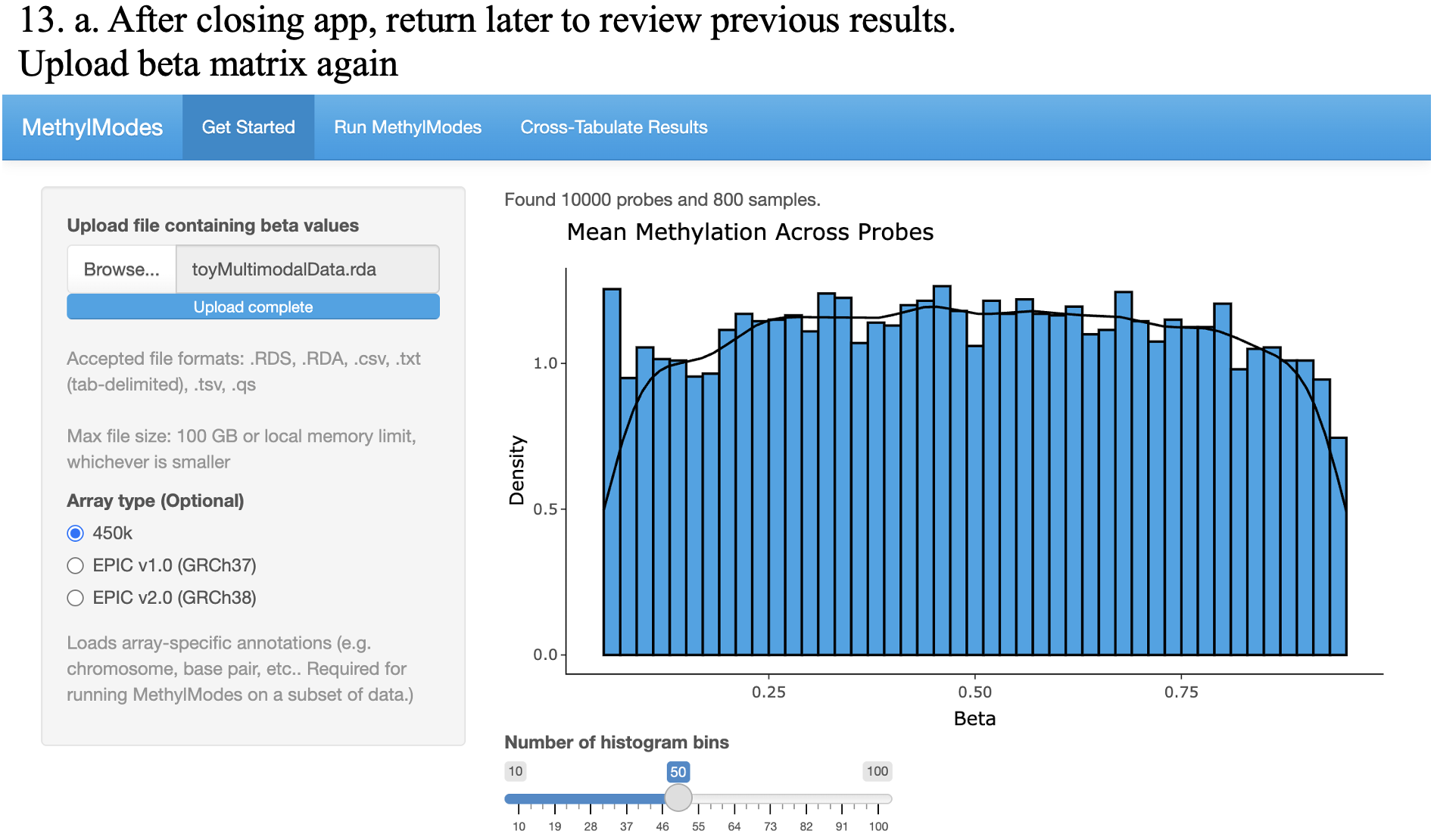

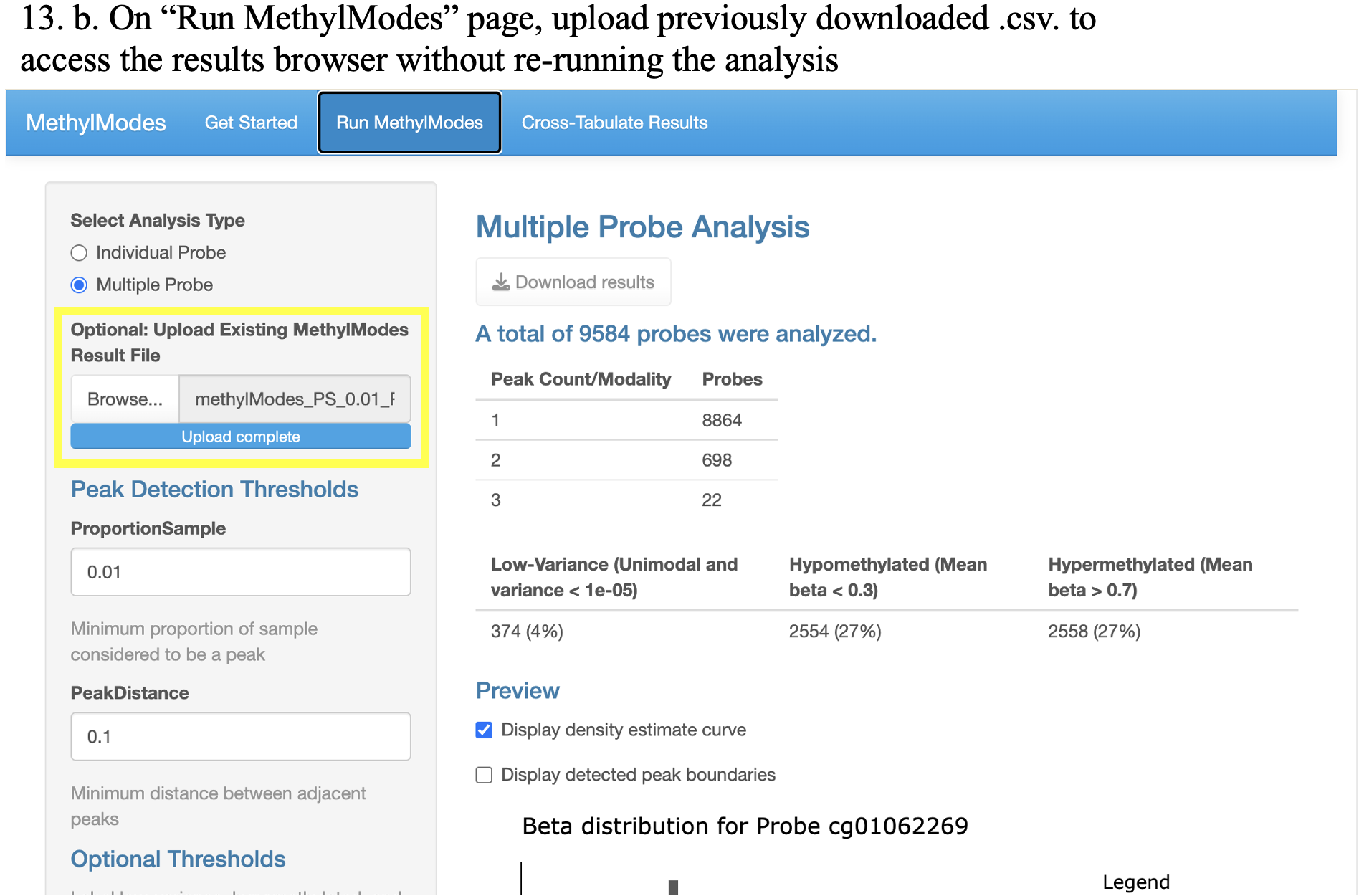
**Supplementary Fig. 1:** Example of a typical MethylModes Shiny app workflow, using the toy dataset included in the R package for demonstration purposes.

INPUT: beta_values # Empirical beta values for one CpG site

PARAMETERS: proportionSample, peakDistance

1. KDE ← stats::density(beta_values) # Smooth beta distribution using kernel density estimation

2. slopes ← diff(KDE.values) # Approximate slope of KDE curve

3. sign_vector ← slopes > 0 # TRUE if slope increases, FALSE otherwise

4. stationary_points ← indices where sign_vector changes (TRUE ↔ FALSE)

5. candidate_peaks ← stationary_points where slope changes from positive to zero/negative

6. IF length(candidate_peaks) == 1 THEN

modality ← "unimodal"

ELSE

# Noise filtering steps

FOR each candidate_peak IN candidate_peaks DO

sample_proportion ← proportion of samples within peak region

IF sample_proportion < proportionSample AND

one bounding minimum == 0 THEN

Remove candidate_peak

END IF

END FOR

# Merge adjacent peaks

merged_peaks ← []

i ← 1

WHILE i ≤ length(candidate_peaks) DO

group ← all candidate_peaks within peakDistance of candidate_peaks[i]

IF length(group) > 1 THEN

merged_peak ← tallest_peak_in(group)

Add merged_peak to merged_peaks

i ← i + length(group)

ELSE

Add candidate_peaks[i] to merged_peaks

i ← i + 1

END IF

END WHILE

# Re-evaluate merged peaks

FOR each peak IN merged_peaks DO

combined_proportion ← proportion of samples within merged peak region

IF combined_proportion < proportionSample THEN

Remove peak from merged_peaks

END IF

END FOR

IF length(merged_peaks) == 1 THEN

modality ← "unimodal"

ELSE

modality ← "multimodal"

END IF

END IF

OUTPUT: modality

**Supplementary Fig. 2:** Pseudocode representation of MethylModes algorithm.

**a.**


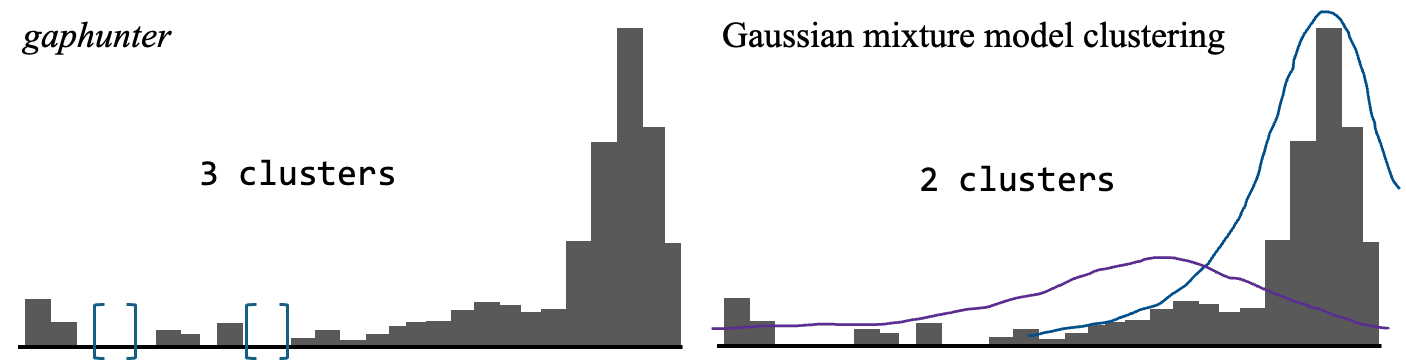


**b.**

**
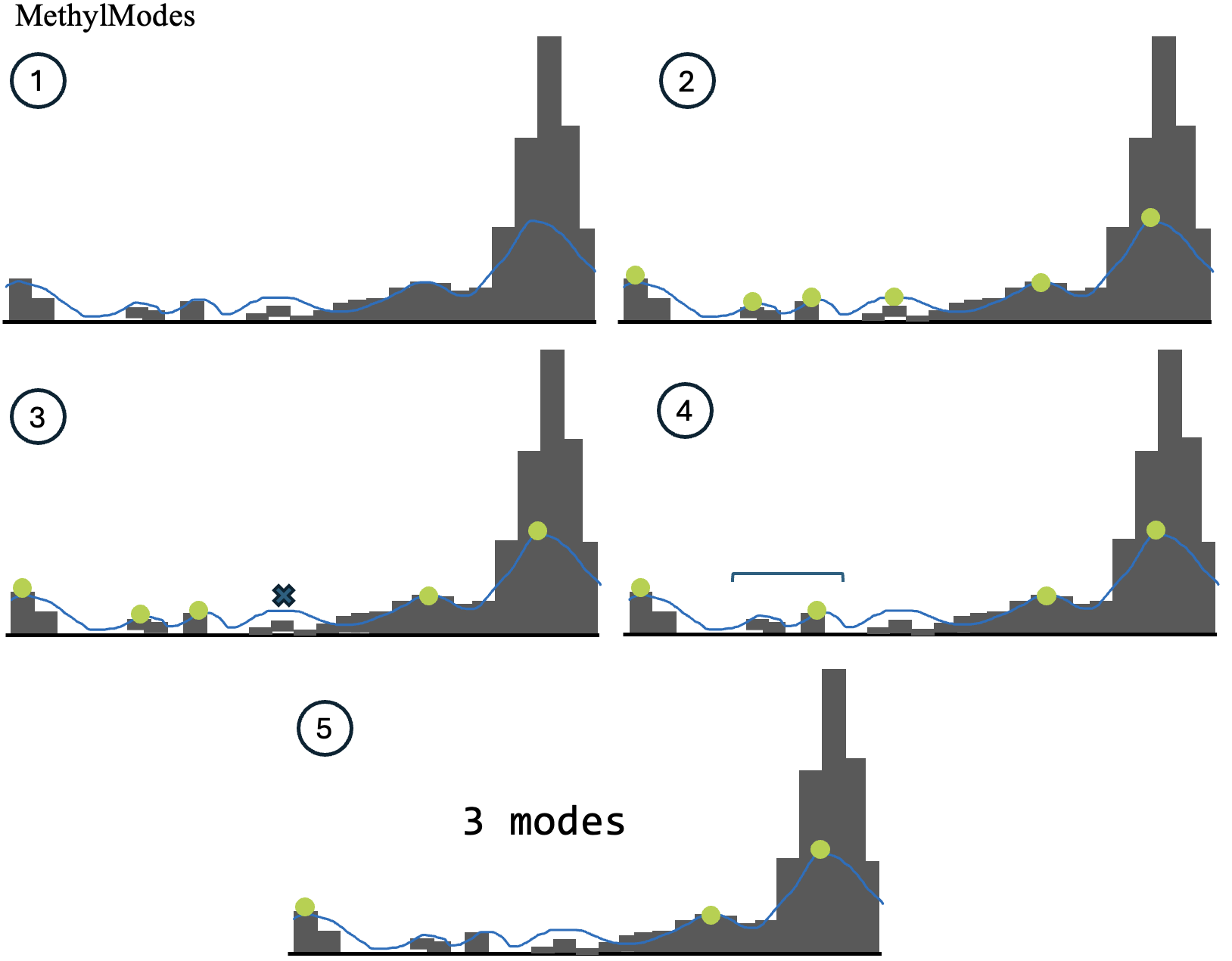
Supplementary Fig. 3:** a**.** Illustration of *gaphunter* and Gaussian mixture model clustering results. b. Illustration of the MethylModes algorithm steps. 1) Perform kernel density estimation; 2) Detect maxima and minima to obtain candidate peaks; 3) Filter by *proportionSample*; 4) Combine peaks less than *peakDistance* apart; 5) Filter new peaks by *proportionSample*; 6) Return remaining peaks.


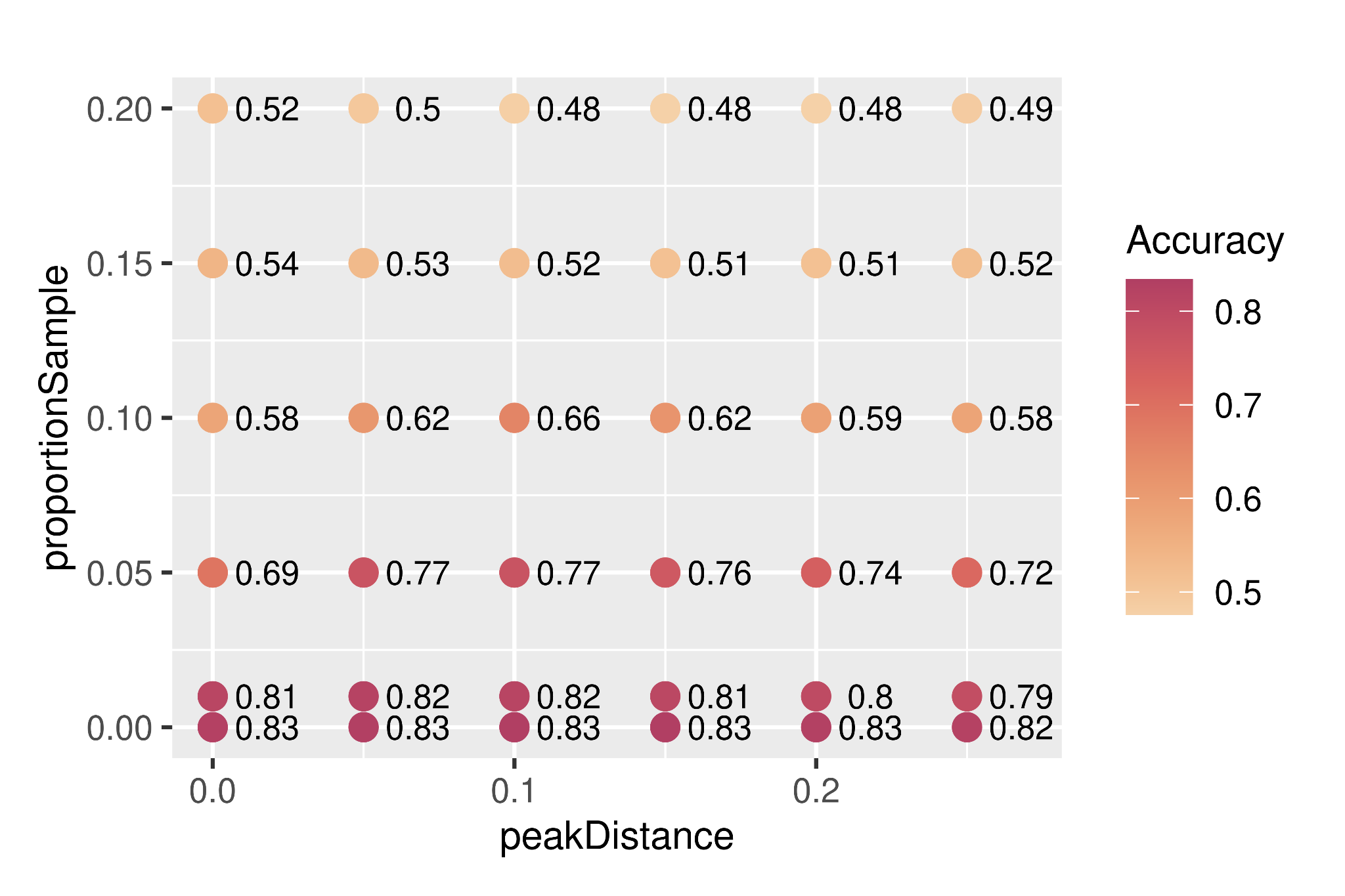


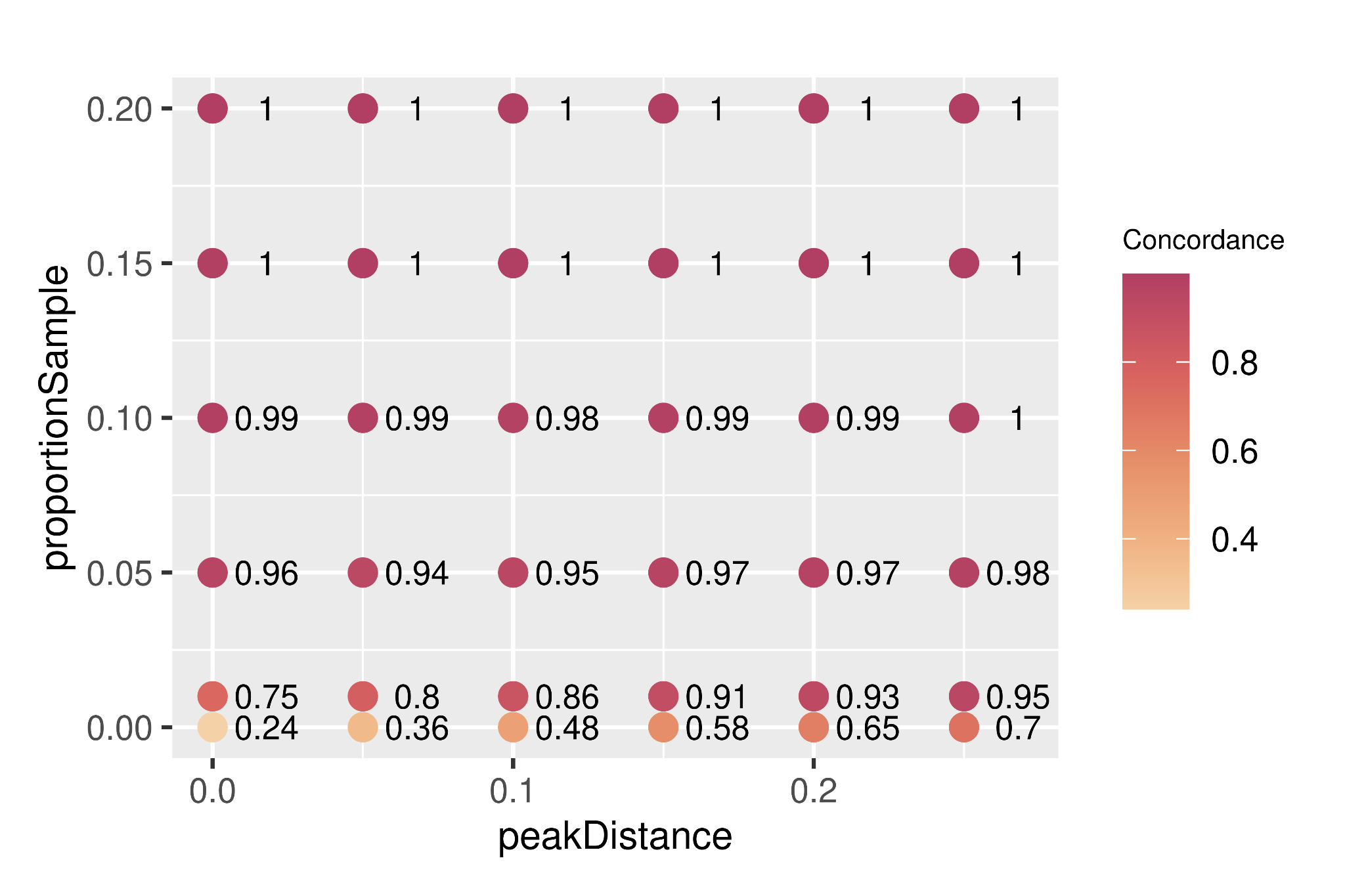


**Supplementary Fig. 4:** Results of grid search used to select MethylModes default parameters. Accuracy is defined as the proportion of MethylModes-called probes that are identified in the Illumina Infinium MethylationEPIC v1.0 BeadChip manifest as overlapping with a genetic variant. The Health and Retirement Study (HRS) sample was randomly split in half and MethylModes was run on all probes using each set of subsamples. Concordance is calculated as the proportion of MethylModes calls that are the same between the two sets of results. To allow for high flexibility in multimodality detection while mitigating the number of false positives due to noise, we selected the smallest values for each parameter that would avoid steep drops in accuracy or concordance. These results suggest that *proportionSample* should be between approximately 0.01 and 0.05, and that *peakDistance* should be at least 0.10. Therefore we set the default MethylModes parameters to the following values: *proportionSample* = 0.01, *peakDistance* = 0.10.


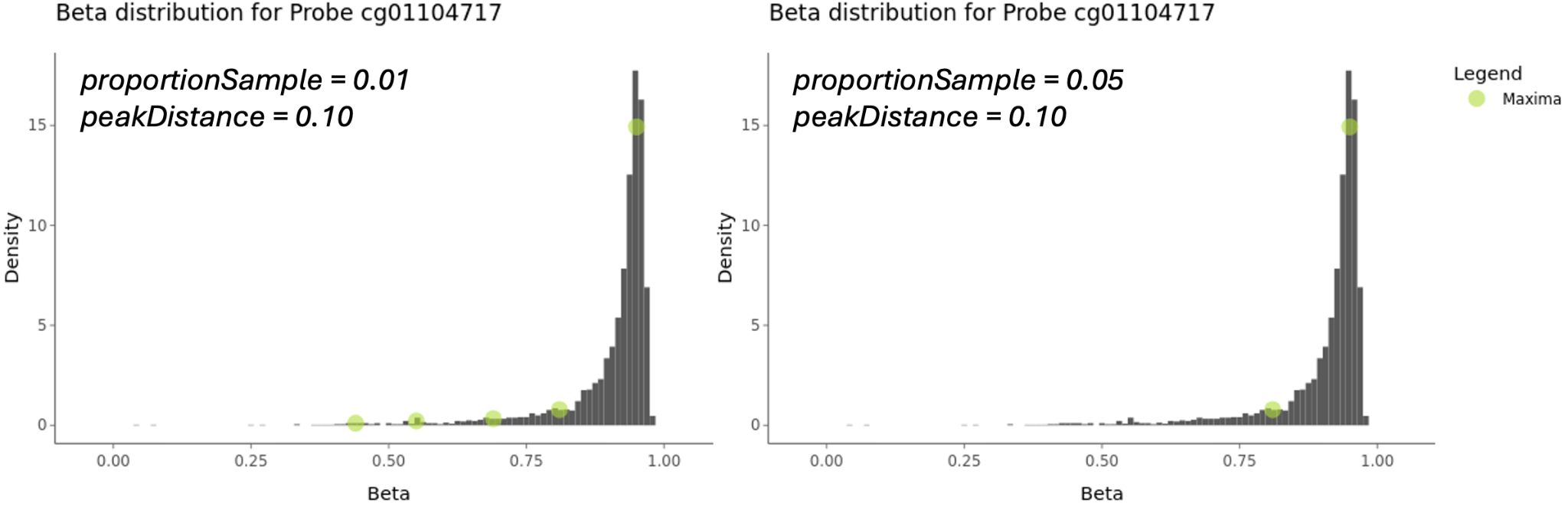


**Supplementary Fig. 5:** Example from Health and Retirement Study (HRS) data of difference in MethylModes-inferred number of modes under different choices of hyperparameter values.

**a.**


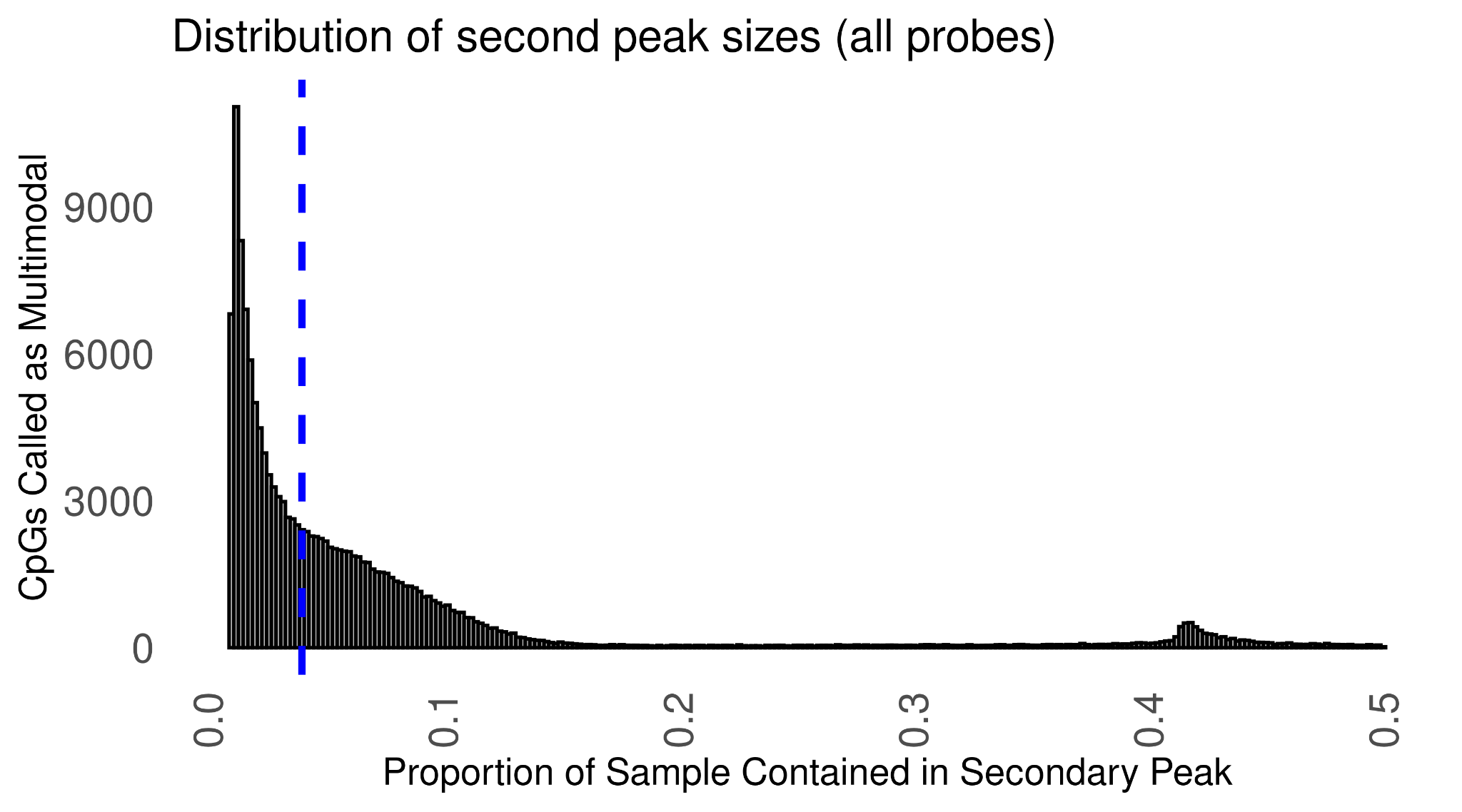


**b.**


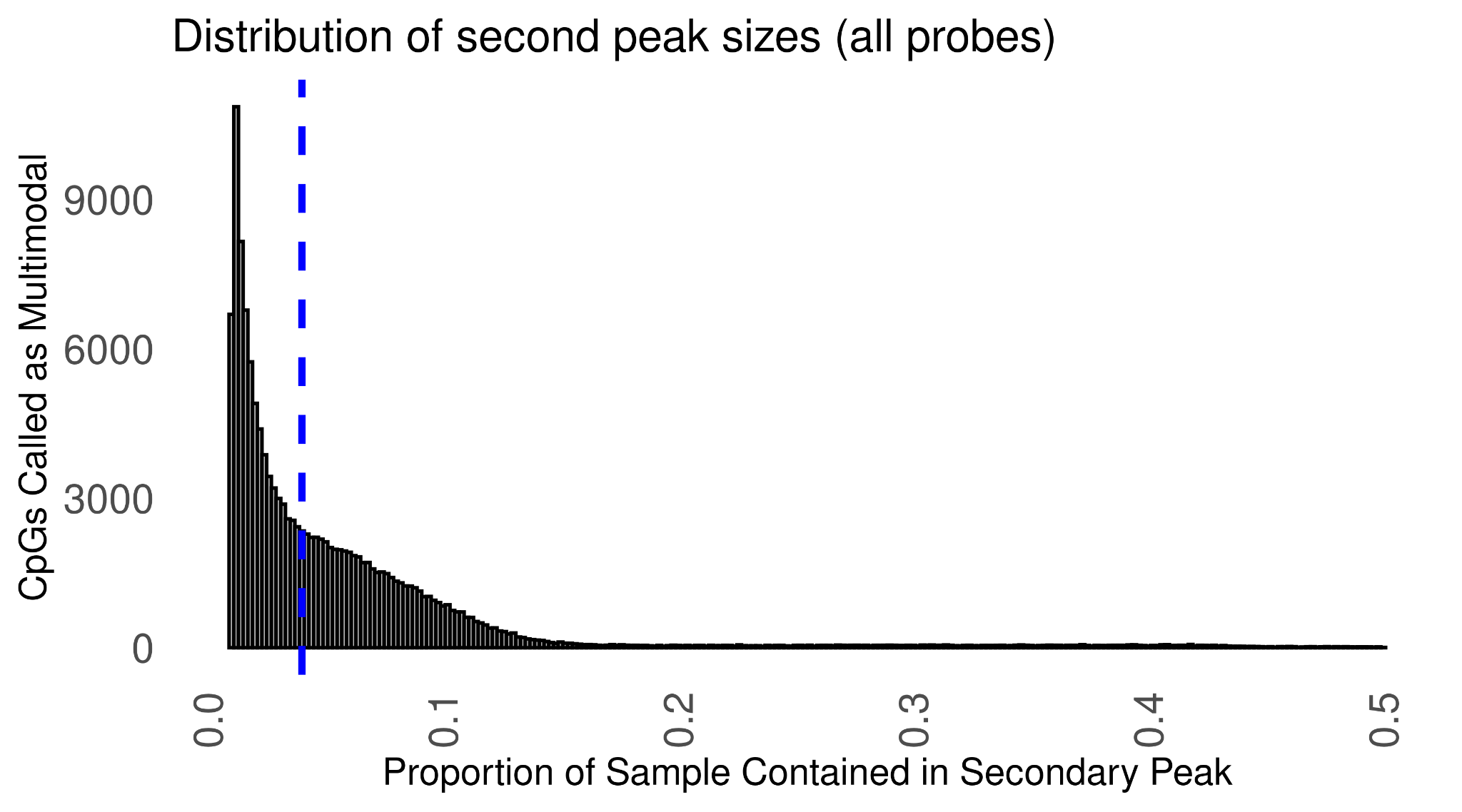


**Supplementary Fig. 6:** a. Distribution of size of the secondary (second-largest) peak for CpG sites called by MethylModes as multimodal. The blue line represents a cutoff point at 0.04 based on visual inspection to exclude low-confidence multimodal calls. b. After excluding CpG sites on the X chromosome, the bump around 0.41 disappears, which is consistent with the multimodal pattern arising from male hemizygosity at X chromosome CpG sites.


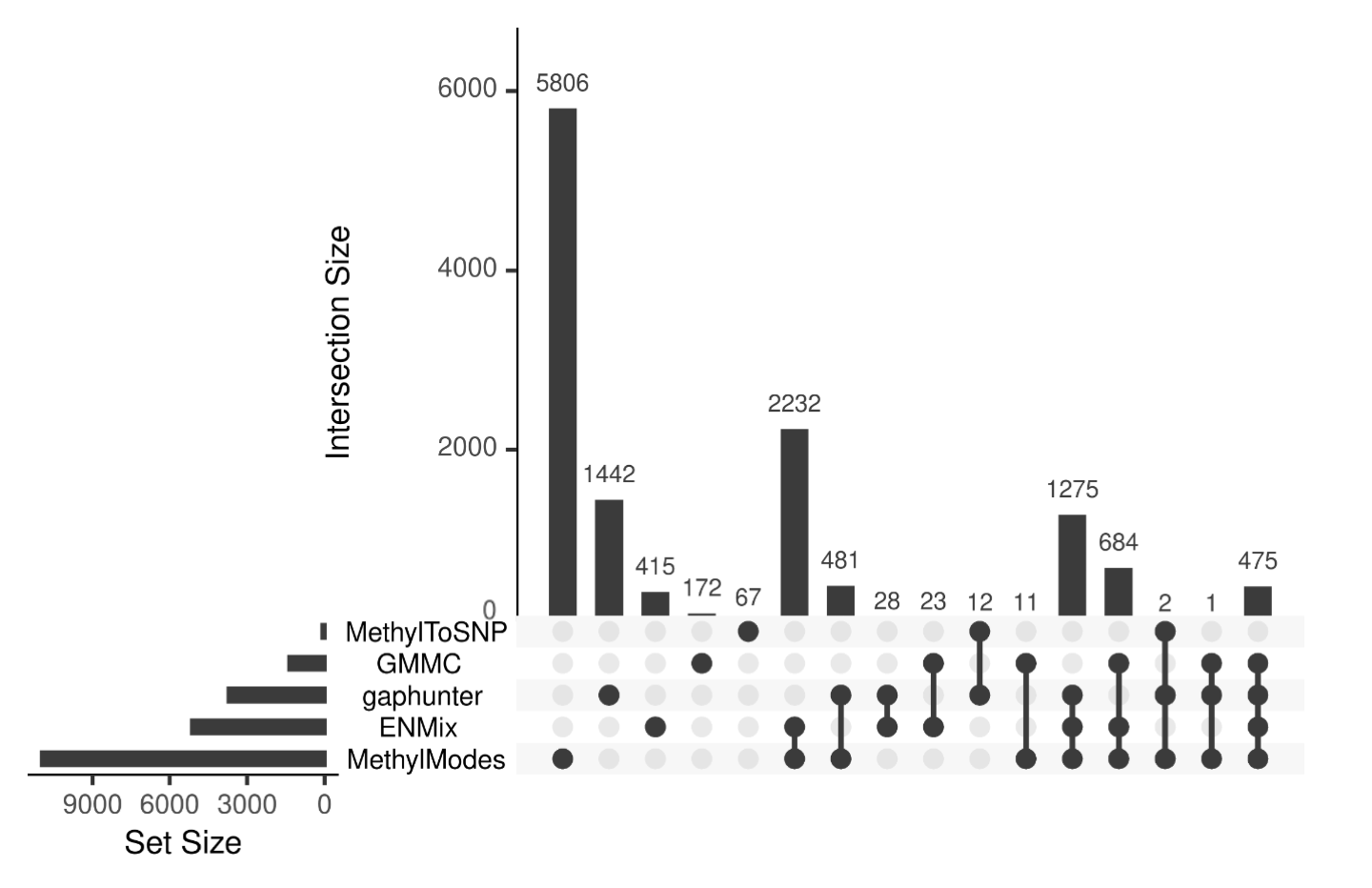


**Supplementary Fig. 7:** Upset plot showing overlap of CpG sites with a variant under the probe (MAF > 0.01) identified by each method as multimodal in the HRS dataset. Probes whose hybridization sequence overlaps with at least one genetic variant under the probe (VUP)

(N = 73,645) were identified using the Illumina Infinium MethylationEPIC v1.0 BeadChip manifest. MethylModes parameters were set to: *proportionSample* = 0.01, *peakDistance* = 0.10. MethylModes results were filtered to remove probes with a secondary peak smaller than 4% of the sample (Supplementary Fig. 6). All other methods were run using author-recommended parameters. MethylModes displayed the highest sensitivity to discovery of CpG sites with VUPs, identifying 10,967 CpG sites with VUPs as multimodal, followed by ENMix (5,132), *gaphunter* (3,716)*,* GMMC (1,366) and finally MethylToSNP (81). As expected, overlap of called multimodal sites was largest between MethylModes and ENMix, based on the similarity of the algorithms, followed by *gaphunter*. MethylToSNP is designed to target a more narrowly defined distribution pattern than other methods, which can be thought of as a special case of the pattern that *gaphunter* targets. Hence, the low number of probes found by MethylToSNP is expected.


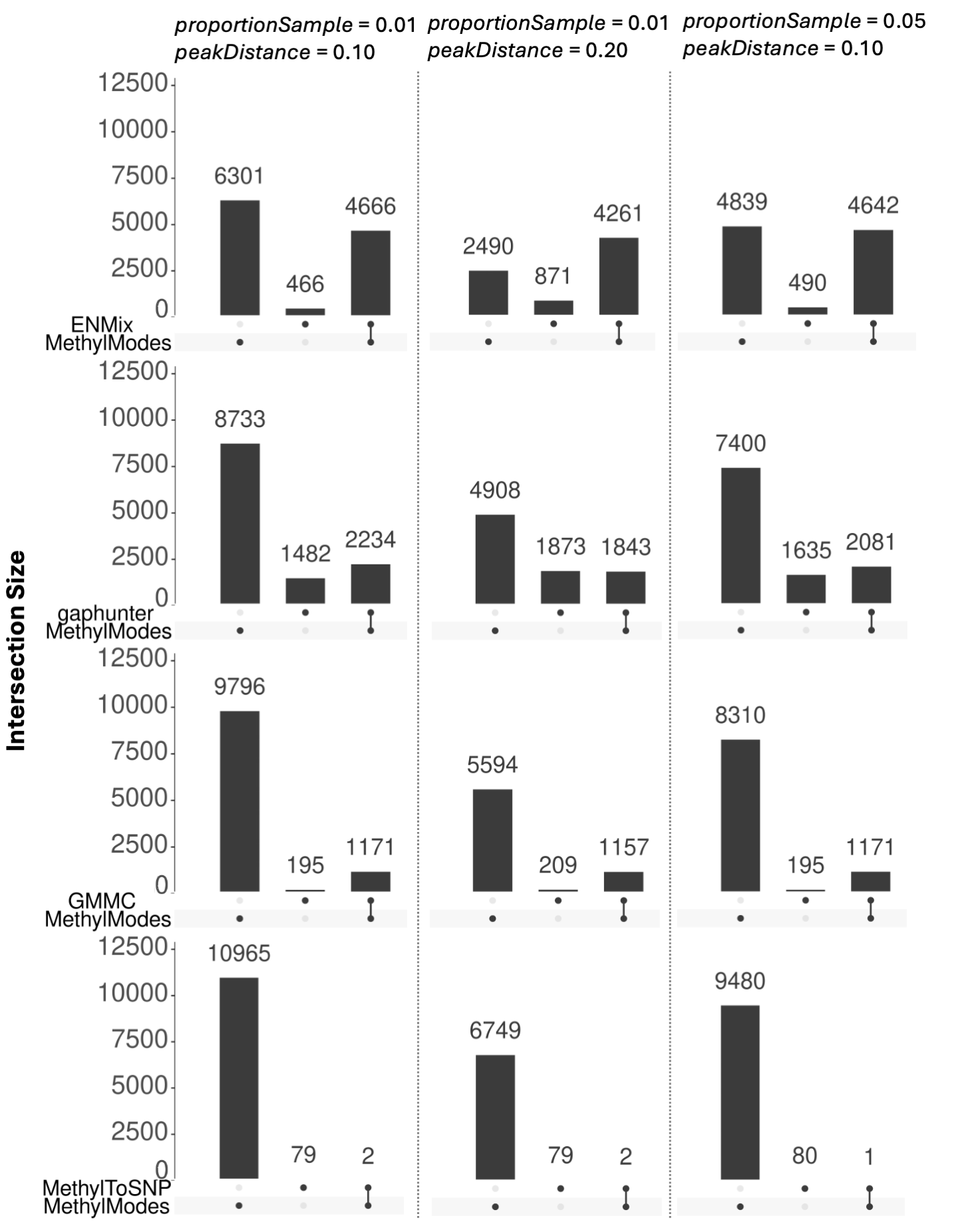


**Supplementary Fig. 8**: Pairwise comparisons between MethylModes and previously existing methods applied to HRS data. All methods other than MethylModes were run using their author-recommended parameters. Across different MethylModes parameter settings, MethylModes consistently demonstrated the greatest sensitivity in calling CpG sites with a variant under the probe as multimodal. The degree of overlap between MethylModes and other methods depends on parameter choice. For example, with *proportionSample* = 0.05 and *peakDistance* = 0.10, the overlap with ENMix results is greater than the overlap under the other parameter choices, but the overlap with *gaphunter* is smaller than it was with *proportionSample* = 0.01 and *peakDistance* = 0.10. In contrast, by setting *proportionSample* = 0.01 leads to greater overlap with *gaphunter*.


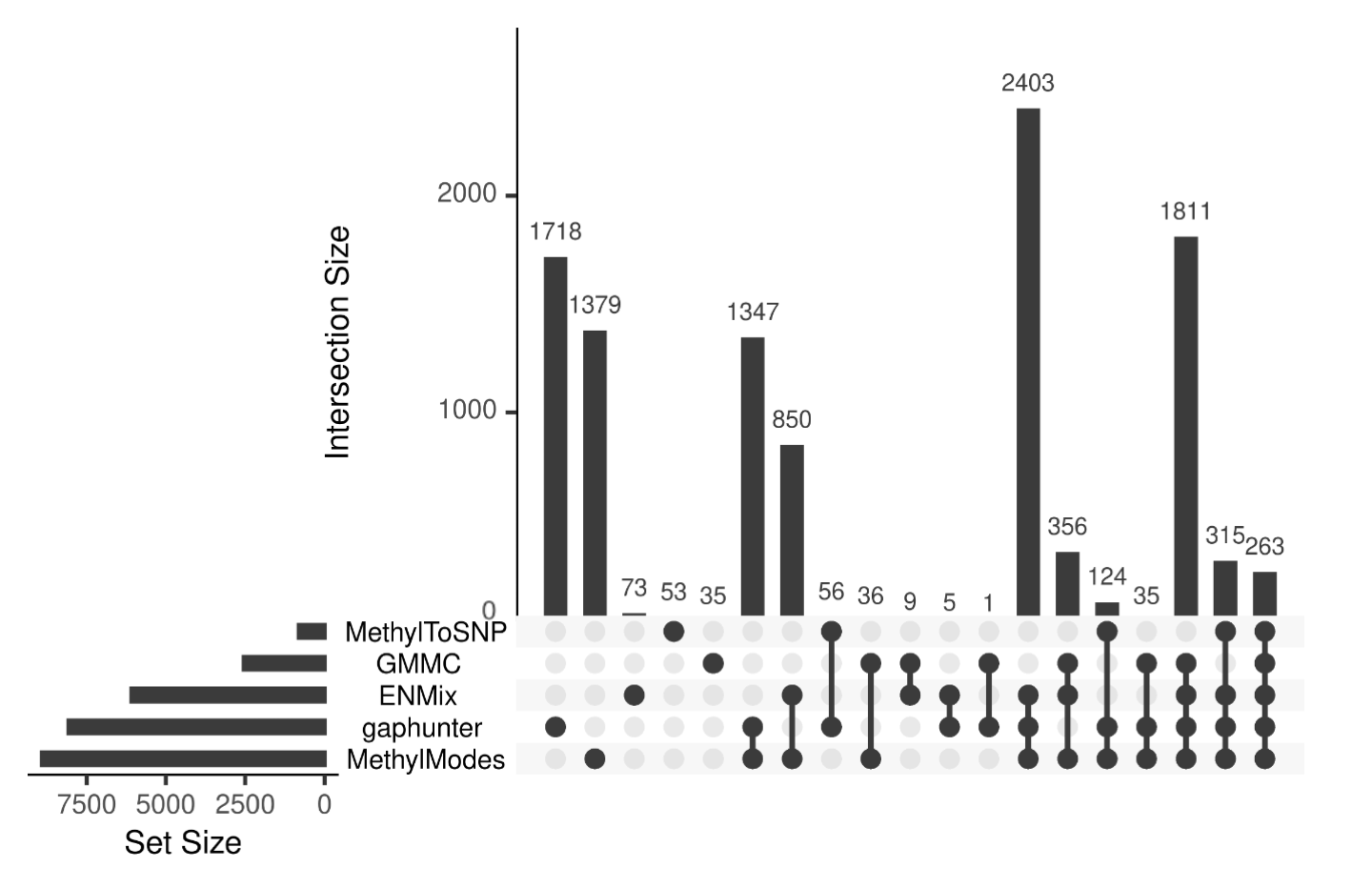


**Supplementary Fig. 9**: Upset plot showing overlap of CpG sites with a variant under the probe (MAF > 0.01) identified by each method as multimodal in the Airwave dataset. Probes whose hybridization sequence overlaps with at least one genetic variant under the probe (VUP)

(N = 73,645) were identified using the Illumina Infinium MethylationEPIC v1.0 BeadChip manifest. MethylModes parameters were set to: *proportionSample* = 0.01, *peakDistance* = 0.10. MethylModes results were filtered to remove probes with a secondary peak smaller than 4% of the sample (Supplementary Fig. 6). All other methods were run using author-recommended parameters. MethylModes displayed the highest sensitivity to discovery of CpG sites with VUPs, identifying 8,919 CpG sites with VUPs as multimodal, followed by *gaphunter* (8,078)*,* ENMix (6,085), GMMC (2,546) and finally MethylToSNP (811). In contrast to the results from HRS data, *gaphunter* had the second highest discovery rate. This finding is consistent with our downsampled results from HRS (Supplementary Fig. 10), which demonstrates that *gaphunter* is much more sensitive when applied to smaller sample sizes.

**a.**


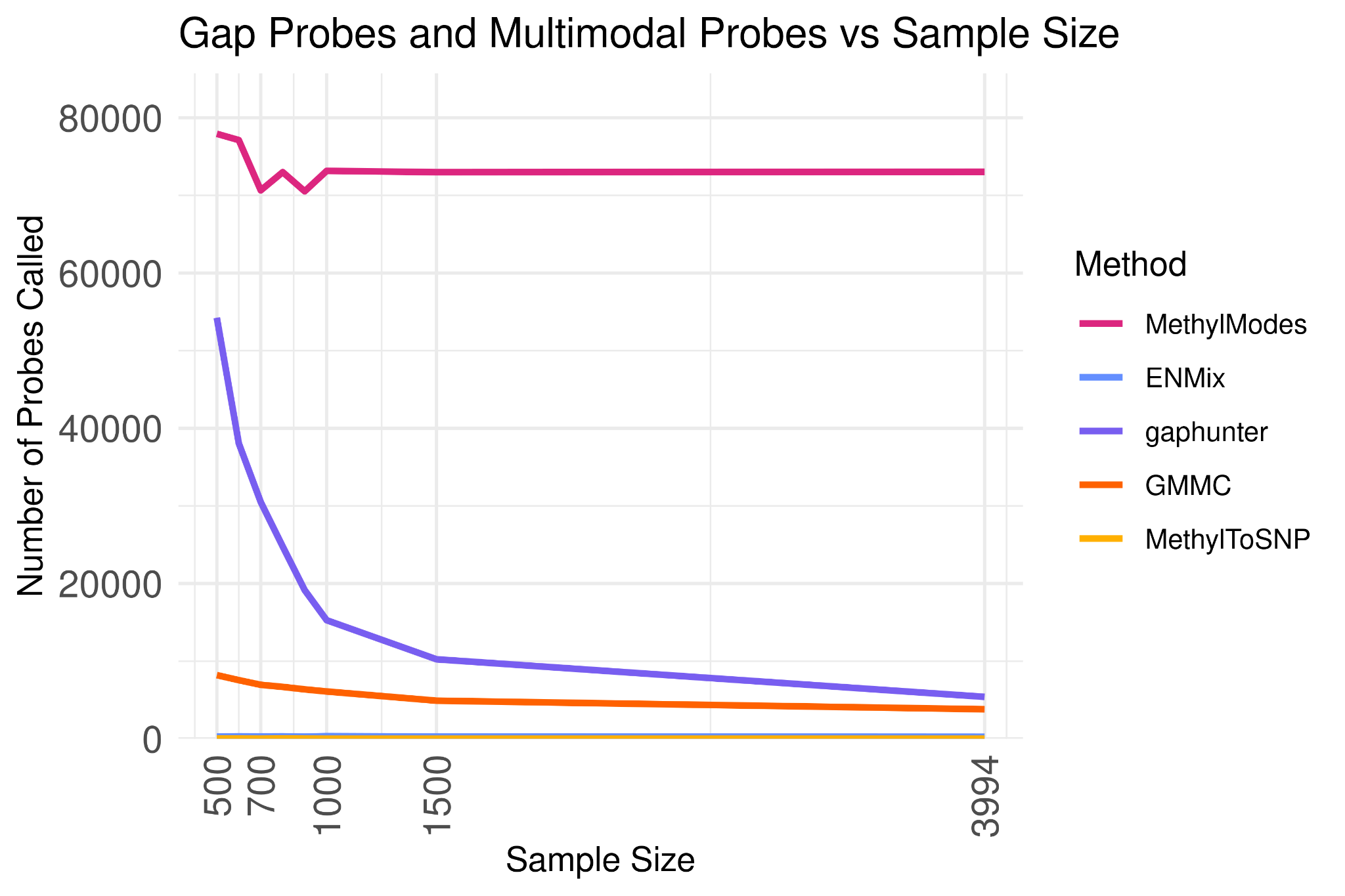


**b.**


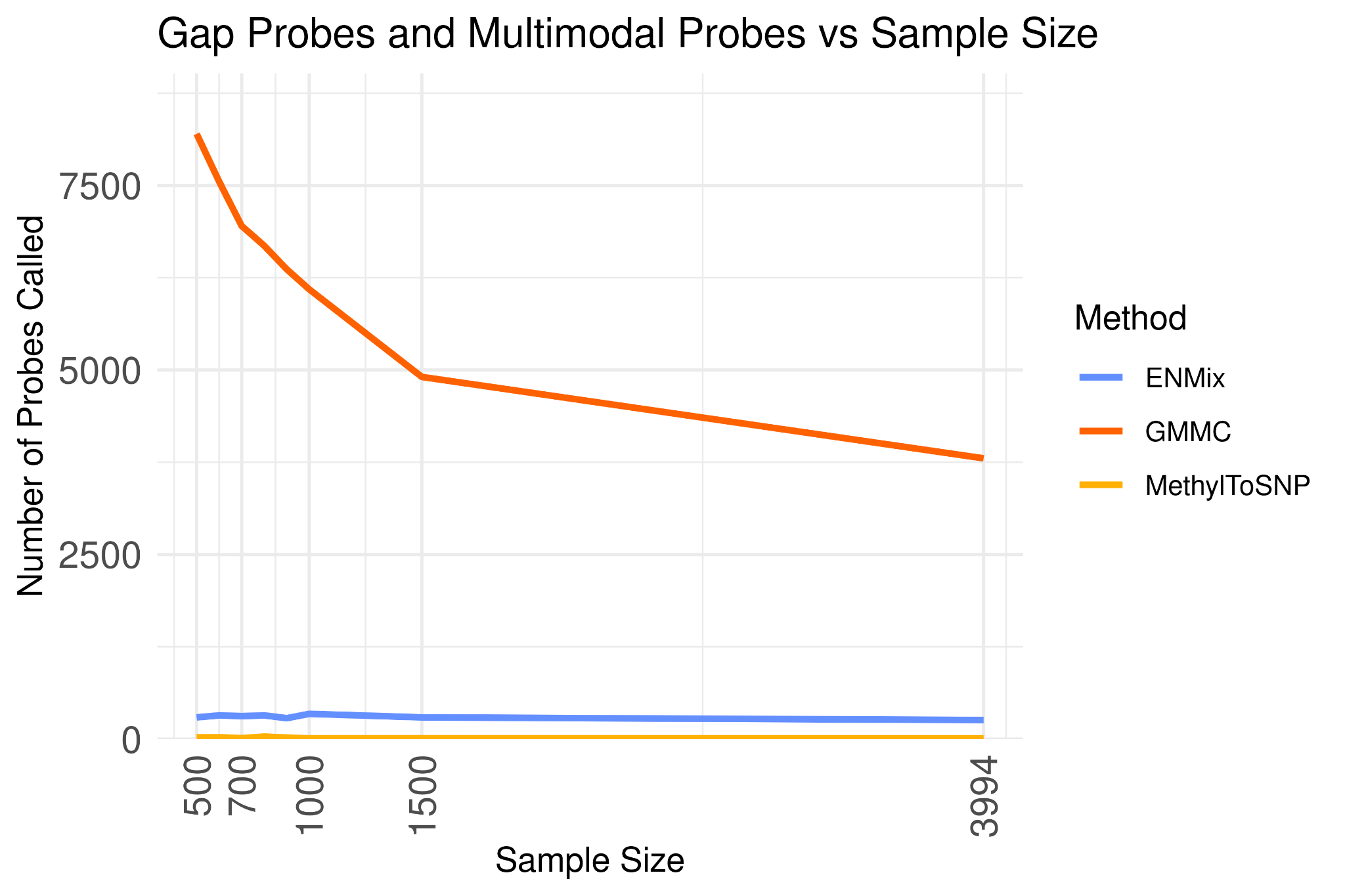


**Supplementary Fig. 10:** a. Comparison of sensitivity to sample size between *gaphunter*, GMMC, and MethylModes. Random subsamples of the HRS sample of size n = 500, 600, 700, 800, 900, 1000, and 1500 were taken to compare the total number of probes called by each method regardless of whether the probe is documented to have a VUP. Results for the full sample size (n = 3994) are also included. MethylModes parameters were set to: *proportionSample* = 0.01, *peakDistance* = 0.10. MethylModes results were filtered to remove probes with a secondary peak smaller than 4% of the sample (Supplementary Fig. 6). All other methods were run using author-recommended parameters. *gaphunter* shows substantially higher sensitivity when run on smaller sample sizes, decreasing in sensitivity as sample size increases. b. Restriction of the plot to ENMix, GMMC, and MethylToSNP for increased visibility.


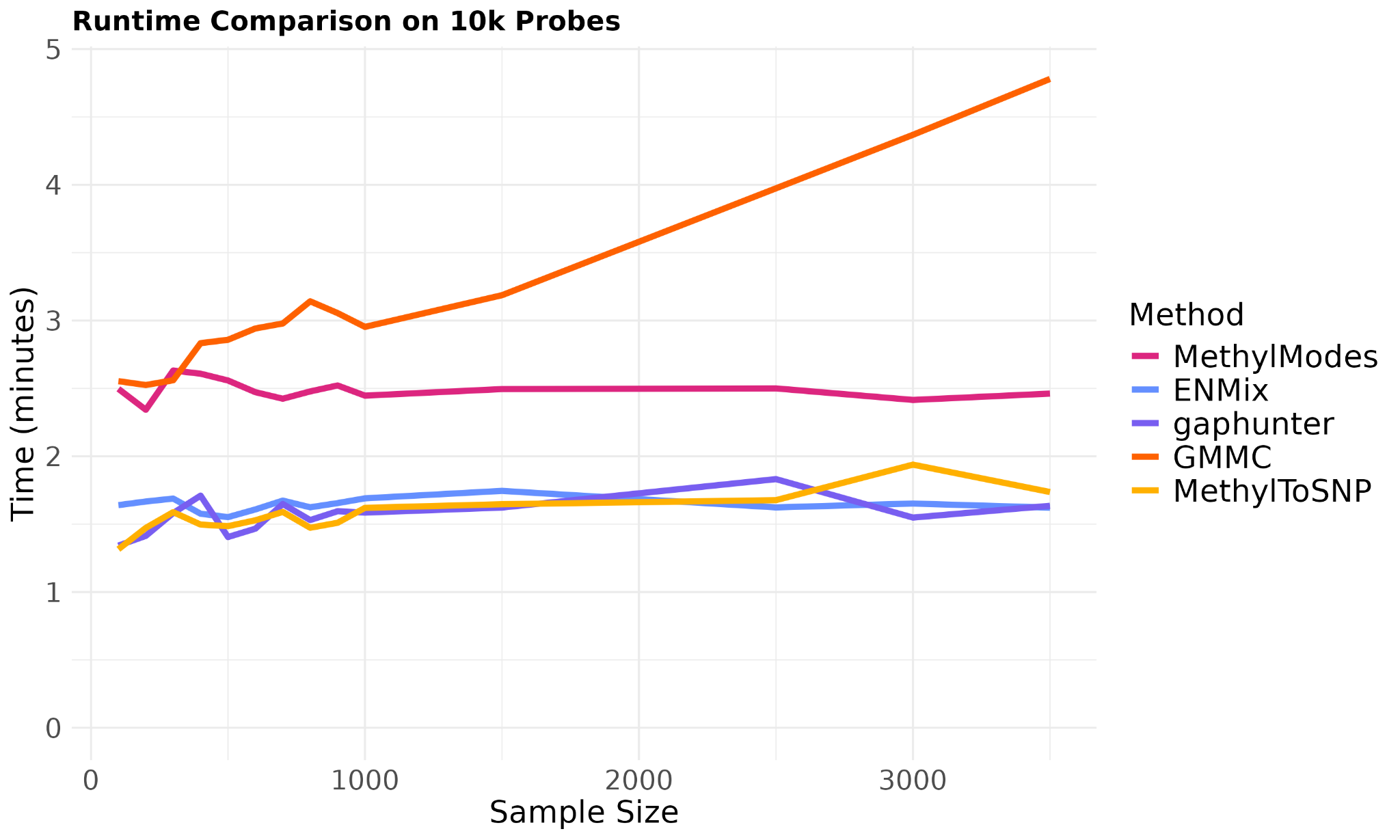


**Supplementary Figure 11**: Runtime comparison between MethylModes and GMMC on a random subset of HRS data of 10,000 probes and a range of sample sizes. Both methods were run 10 times at each sample size. GMMC runtime steadily increases in an approximately linear fashion as a function of sample size, while virtually no change is observed in the other methods. MethylModes runtime is generally faster than that of GMMC for sample sizes greater than approximately 500, while it remains slower than ENMix, *gaphunter*, and MethylToSNP by a constant factor.
